# Phenolic Profile and Antioxidant Potential of Selected European *Astragalus* Species: Comparative UHPLC–DAD–ESI/TOF–MS and In Vitro Study

**DOI:** 10.3390/antiox15060750

**Published:** 2026-06-13

**Authors:** Jakub Gębalski, Milena Gębalska, Ewa Kiełkowska, Piotr Sit, Iga Hołyńska-Iwan, Magdalena Wójciak, Daniel Załuski

**Affiliations:** 1 Department of Pharmaceutical Botany and Pharmacognosy, Ludwik Rydygier Collegium Medicum, Nicolaus Copernicus University, 85-094 Bydgoszcz, Poland; milena.malkowska@cm.umk.pl (M.G.); ewa.kielkowska@cm.umk.pl (E.K.); daniel.zaluski@cm.umk.pl (D.Z.); 2 Warsaw Medical and Technical Academy of Applied Sciences, ul. Bitwy Warszawskiej 1920 18, 02-366 Warsaw, Poland; p.sit@interia.pl; 3 Department of Pathobiochemistry and Clinical Chemistry, Ludwik Rydygier Collegium Medicum, Nicolaus Copernicus University, 85-094 Bydgoszcz, Poland; igaholynska@cm.umk.pl; 4 Department of Analytical Chemistry, Medical University of Lublin, 20-093 Lublin, Poland; magdalena.wojciak@umlub.edu.pl

**Keywords:** *Astragalus membranaceus*, *Astragalus cicer*, *Astragalus glycyphyllos*, phenolic compounds, DPPH, ABTS, CUPRAC, FRAP, ion chelation, superoxide anion, α-amylase, hyaluronidase, lipase, lipid peroxidation, UHPLC–DAD–ESI/TOF–MS, TPC, TFC, TPAC

## Abstract

Plants of the genus *Astragalus* are recognized as rich sources of bioactive compounds with antioxidant and therapeutic potential; however, European species remain less explored than the well-known *Astragalus membranaceus* (Fisch.) Bunge. The aim of this study was to compare the phytochemical composition and in vitro biological activity of selected *Astragalus* species occurring in Poland (*A. cicer* L., *A. glycyphyllos* L., *A. membranaceus*). Phenolic compounds in methanolic extracts obtained from the roots and aerial parts were analyzed using spectrophotometric methods and UHPLC–DAD–ESI/TOF–MS. Antioxidant activity was evaluated using DPPH, ABTS, FRAP, CUPRAC, metal chelation, superoxide radical scavenging, and lipid peroxidation (TBARS) assays. Additionally, enzyme inhibition toward α-amylase, lipase, hyaluronidase, tyrosinase, and butyrylcholinesterase was assessed. The root of *A. membranaceus* exhibited the highest total phenolic content (199.84 ± 3.64 mg GAE/g extract) and the strongest antioxidant activity (DPPH IC_50_ = 36.53 ± 1.22 µg/mL; ABTS IC_50_ = 26.31 ± 0.03 µg/mL), as well as the most pronounced α-amylase inhibition (IC_50_ = 17.78 ± 1.16 µg/mL). It also demonstrated moderate protective effects against AAPH-induced lipid peroxidation. The herb of *A. cicer* showed moderate radical scavenging capacity and the most effective inhibition of lipid peroxidation at higher concentrations. Extracts of *A. glycyphyllos* displayed weaker radical scavenging but notable metal-chelating properties. Selected extracts also exhibited moderate inhibitory activity against tyrosinase and butyrylcholinesterase. *A. membranaceus* remains the most potent source of phenolic compounds and antioxidant activity; European species such as *A. cicer* and *A. glycyphyllos* represent promising, locally available alternatives and may be used in phytotherapy and functional products.

## 1. Introduction

Plants of the genus *Astragalus* L. are characterized by a broad spectrum of biological activity, as confirmed by centuries of traditional medicinal use as well as contemporary scientific research [1]. The genus belongs to the Fabaceae family and comprises approximately 2000–3000 species distributed across different continents, making it one of the largest and most diverse genera within this family [2]. Many *Astragalus* species have been used in traditional medicine for nearly two thousand years, particularly in Asia, where they have been applied in the treatment of various conditions, including respiratory tract infections, diabetes and leukemia [3].

The therapeutic properties of *Astragalus* species result from their rich phytochemical composition, which includes numerous well-characterized biologically active compounds. Among the polysaccharides, fractions designated as APS I, APS II, APS III and APS IV have been isolated. These are heteropolysaccharides composed mainly of glucose, arabinose, galactose and rhamnose. A particularly important group consists of triterpenoid saponins with a cycloartane-type skeleton, among which astragalosides I, II, III and IV are the most extensively studied, along with isoastragalosides I and II and acetylastragaloside I. Key flavonoids identified in species of this genus include formononetin, calycosin, calycosin-7-O-*β*-D-glucoside, ononin, isorhamnetin, kaempferol and quercetin [4,5,6].

Among the representatives of the genus, *Astragalus membranaceus* (Fisch.) Bunge (AM), commonly known as Huangqi or Mongolian milkvetch, is of particular importance. This species has long occupied a prominent position in natural pharmacology, especially in traditional Chinese medicine, where it is valued for its immunostimulatory and tonic properties [7]. The root of AM contains numerous active constituents, including high-molecular-weight polysaccharides, flavonoids and triterpenoid saponins (astragalosides). These compounds are responsible for a wide range of beneficial pharmacological effects, demonstrating immunomodulatory, anti-inflammatory and anticancer activities. The plant is also regarded as a classical adaptogen that enhances the body’s resistance to stress [8]. Among its active components, astragaloside IV, the principal saponin constituent, has attracted particular attention due to its pleiotropic activity, including anticancer, anti-inflammatory, cardioprotective and immunomodulatory effects demonstrated in experimental studies [9].

In Europe, native *Astragalus* species such as *Astragalus cicer* L. (AC) and *Astragalus glycyphyllos* L. (AG) also occur; however, they are less recognized and less studied than AM. Nevertheless, they have also been used in folk medicine. For example, *A. glycyphyllos* has been employed in Bulgarian traditional medicine as an antihypertensive, diuretic and anti-inflammatory agent, whereas *A. cicer* has been administered in Belarusian folk medicine for heart diseases and gastrointestinal disorders [10,11]. To date, scientific reports concerning their chemical composition and pharmacological activity remain limited and fragmented, and comprehensive comparative analyses are lacking. Therefore, the main objective of the present study was to evaluate whether AC and AG exhibit a comparable phytochemical profile and similar biological activity, which could justify their potential use as substitutes for AM in phytotherapy.

## 2. Materials and Methods

### 2.1. Preparation of Raw Material

The aerial parts and roots of three species from the genus *Astragalus* were used in the study: *Astragalus membranaceus* (Fisch.) ex Bunge, *Astragalus glycyphyllos* L., and *Astragalus cicer* L. *Astragalus cicer* and *Astragalus glycyphyllos* were collected from natural populations in Poland. *A. cicer* was collected in Kórnik, Poland (52°17′10.0″ N, 17°04′19.5″ E), while *A. glycyphyllos* was collected in Nowy Duninów, Poland (52°34′27.7″ N, 19°26′52.0″ E). *A. membranaceus* was cultivated from seeds obtained from Adaptogeny Rzeszów, Handzlówka, Poland, and grown in the medicinal plant garden of the Faculty of Pharmacy, Collegium Medicum, Nicolaus Copernicus University in Bydgoszcz, Poland (53°07′36.8″ N, 18°01′51.3″ E). The aerial parts of all studied species were collected in July 2023, whereas the roots were collected in October 2023. The identity of all plant materials was confirmed on the basis of morphological characteristics by Prof. Daniel Załuski. Following collection, the raw materials were freeze-dried using an Alpha 1–2 LDplus freeze dryer (Martin Christ, Osterode am Harz, Germany) and stored at −20 °C until extraction and further analyses. AM, AC, and AG refer to *Astragalus membranaceus*, *Astragalus cicer*, and *Astragalus glycyphyllos*, respectively. The suffixes R and H indicate root and herb, respectively. Accordingly, AMR and AMH refer to the root and herb of *A. membranaceus*, ACR and ACH to the root and herb of *A. cicer*, and AGR and AGH to the root and herb of *A. glycyphyllos*.

### 2.2. Preparation of Extract

The extracts were prepared using an ultrasound-assisted maceration method. Ten grams of plant material were extracted with 100 mL of 75% (*v*/*v*) methanol. The mixture was incubated at room temperature (22 ± 2 °C) for 24 h. After maceration, the extract was subjected to sonication (Polsonic Sonic 2, 40 kHz, Warsaw, Poland) for 15 min at room temperature. The extract was then filtered and concentrated under reduced pressure at 30 °C, using a rotary evaporator (Büchi Heating Bath B-490 and Rotavapor R-200, Büchi Labortechnik AG, Flawil, Switzerland), followed by freeze-drying at −20 °C (Alpha 1–2 LDplusMartin Christ Osterode am Harz, Germany). The obtained dry extracts were stored at −20 °C until further analysis.

### 2.3. Reagents

All reagents used in this study were of analytical grade. Nitro blue tetrazolium chloride (NBT), xanthine, xanthine oxidase, 2,2-diphenyl-1-picrylhydrazyl (DPPH), 2,2′-azinobis-(3-ethylbenzthiazoline-6-sulfonic acid) (ABTS), potassium persulfate, 3-(2-pyridyl)-5,6-diphenyl-1,2,4-triazine-p,p′-disulfonic acid monosodium salt hydrate (ferrozine), iron(II) chloride tetrahydrate (FeCl_2_·4H_2_O), iron(III) chloride (FeCl_3_), 1,3,5-tri(2-pyridyl)-2,4,6-triazine (TPTZ), copper(II) chloride (CuCl_2_), neocuproine, aluminum chloride (AlCl_3_), potassium acetate, sodium carbonate, Folin–Ciocalteu reagent, sodium nitrite, sodium molybdate, hydrochloric acid, sodium hydroxide, acetic acid, Trolox, ascorbic acid, butylated hydroxyanisole (BHA), ethylenediaminetetraacetic acid (EDTA), thiobarbituric acid (TBA), trichloroacetic acid (TCA), 2,2′-azobis(2-amidinopropane) dihydrochloride (AAPH), hyaluronidase from bovine testes, hyaluronic acid, cetyltrimethylammonium bromide (CTAB), bovine serum albumin (BSA), α-amylase, soluble starch, iodine solution, pancreatic lipase, p-nitrophenyl dodecanoate (p-NPD), tyrosinase, L-tyrosine, butyrylcholinesterase, 5,5′-dithiobis-(2-nitrobenzoic acid) (DTNB), escin, acarbose, orlistat, kojic acid, donepezil, gallic acid, quercetin, and caffeic acid were purchased from Sigma-Aldrich Corp. (St. Louis, MO, USA).

The following reagents were used for buffer preparation: sodium chloride (NaCl), potassium chloride (KCl), disodium hydrogen phosphate (Na_2_HPO_4_), potassium dihydrogen phosphate (KH_2_PO_4_), sodium phosphate monobasic (NaH_2_PO_4_), sodium phosphate dibasic (Na_2_HPO_4_), sodium acetate (CH_3_COONa), glacial acetic acid (CH_3_COOH), tris(hydroxymethyl)aminomethane (Tris), and hydrochloric acid (HCl). All reagents were of analytical grade and were obtained from Sigma-Aldrich (St. Louis, MO, USA).

Chromatographic standards, such as astragaloside I, astragaloside II, astragaloside III, astragaloside IV and selected isoflavonoids characteristic of the genus *Astragalus*, namely, calycosin-7-O-*β*-D-glucoside, formononetin-7-O-*β*-D-glucopyranoside, calycosin and formononetin, were purchased from MedChemExpress (Monmouth Junction, NJ, USA).

Solvents used for extraction were purchased from Avantor Performance Materials (Gliwice, Poland).

All chemicals used for chromatographic analyses, including analytical standards, formic acid, and acetonitrile of mass spectrometry grade, were purchased from Sigma-Aldrich (St. Louis, MO, USA).

### 2.4. Phytochemical Characterization

#### 2.4.1. Total Polyphenol Content (TPC)

The total phenolic content of the extract was determined using the Folin–Ciocalteu (FC) colorimetric method, with slight modifications [12]. The assay was conducted in 96-well microplates. Briefly, 25 μL of the test extract (1.0 mg/mL in 50 mM PBS, pH 7.4), 25 μL of a 3-fold diluted FC reagent, and 200 μL of distilled water were added to each well. The plate was then incubated for 5 min at room temperature. Subsequently, 25 μL of a saturated sodium carbonate (10% in distilled water) solution was added to each well, and the reaction mixture was incubated in the dark at room temperature for 60 min. After incubation, the absorbance of each sample was measured at 750 nm using a microplate reader. All measurements were performed in triplicate. The total phenolic content was quantified based on a gallic acid standard calibration curve and expressed as milligrams of gallic acid equivalents per gram of dry extract (mg GAE/g DE).

#### 2.4.2. Total Flavonoids Content (TFC)

The total flavonoid content of the extract was determined using the aluminum chloride colorimetric method [13]. In a 96-well microplate, 25 μL of the extract (1.0 mg/mL in 50 mM PBS, pH 7.4), 75 μL of ethanol, 10 μL of aluminum chloride solution (10% in distilled water, AlCl_3_), 10 μL of potassium acetate solution (1M in distilled water), and 130 μL of distilled water were added to each well. The reaction mixtures were incubated at room temperature for 30 min in the dark to prevent photodegradation. After incubation, absorbance was measured at 510 nm using a microplate reader. All samples were analyzed in triplicate to ensure data reliability. The flavonoid content was quantified based on a quercetin calibration curve and expressed as milligrams of quercetin equivalents per gram of dry extract (mg QE/g DE).

#### 2.4.3. Total Phenolic Acid Content (TPAC)

The total phenolic acid content was determined using Arnov’s reagent colorimetric method [14]. In a 96-well microplate, 25 μL of the extract (1.0 mg/mL in 50 mM PBS, pH 7.4), 150 μL of distilled water, 25 μL of hydrochloric acid solution (0.5M in distilled water), 25 μL of Arnov’s reagent (10.0 g of sodium molybdate and 10.0 g of sodium nitrite in 100 mL distilled water), and 25 μL of sodium hydroxide solution (1M in distilled water) were sequentially added to each well. The absorbance of the resulting solutions was measured at 492 nm using a microplate reader. All measurements were performed in triplicate for accuracy and reproducibility. The phenolic acid content was calculated based on a calibration curve prepared with caffeic acid and expressed as milligrams of caffeic acid equivalents per gram of dry extract (mg CAE/g DE).

#### 2.4.4. Chromatographic Analysis

Analytical measurements were performed using an Infinity II Series Ultra-High-Performance Liquid Chromatography (UHPLC) system equipped with a diode array detector (DAD) and coupled with an Agilent 6224 Electrospray Ionization Time-of-Flight (ESI/TOF) mass spectrometer (Agilent Technologies, Santa Clara, CA, USA). Chromatographic separation was carried out on a Kinetex C18 reversed-phase column (Phenomenex, Torrance, CA, USA; 150 mm × 2.1 mm, 1.7 µm particle size, 100 Å). The column was maintained at a constant temperature of 30 °C, with the mobile phase delivered at a flow rate of 0.2 mL/min. The mobile phase consisted of solvent A (water containing 0.05% formic acid) and solvent B (acetonitrile with 0.05% formic acid). Gradient elution for phenolic compounds was performed as follows: 0–8 min from 98% A to 93% A, 8–15 min from 93% A to 88% A, 15–29 min from 88% A to 85% A, 29–40 min from 85% A to 80% A, 40–60 min from 80% A to 65% A, and 60–85 min from 65% A to 50% A. UV-Vis spectra were recorded over the wavelength range of 195–600 nm. Semiquantitative analysis was performed using UV detection based on an external standard calibration method. For high-molecular-weight flavonoids and derivatives of phenolic acids, quantification was carried out based on calibration curves for compounds containing the appropriate aglycone. The calculated concentrations based on aglycone calibration curves were converted to the corresponding glycoside equivalents by applying a molecular weight correction factor. Mass spectrometric analysis for phenolics compounds was performed in negative ionization mode under the following operating parameters: drying gas temperature 325 °C, gas flow rate 8 L/min, nebulizer pressure 30 psi, capillary voltage 3500 V, skimmer voltage 65 V, and fragmentor voltage set at 200, 280 and 320 V. Positive ionization at 140V was applied for astragalosides. The mass spectra were acquired in a scan range of *m*/*z* 100–1200. For isoflavones, quantification was carried out using extracted ion chromatograms (EIC) obtained from High-Resolution Mass Spectrometry (HRMS) data.

### 2.5. Biological Panel

#### 2.5.1. Enzymatic Assays

All test samples were dissolved in buffer prior to analysis. The extracts were evaluated at concentrations ranging from 0.01 to 10 mg/mL. The half-maximal inhibitory concentration (IC_50_) values were calculated by applying linear regression analysis to the dose–response data, using the equation y = ax + b, where y represents the percentage of inhibition and x denotes the concentration of the extract.

Hyaluronidase Inhibitory Assay

The hyaluronidase inhibitory activity of the tested compounds and extracts was evaluated using a microplate-based method adapted from Di Ferrante [15] and Studzińska-Sroka [16], with minor modifications [17]. The assay was carried out in 96-well plates and relied on the precipitation of undigested hyaluronic acid (HA) by cetyltrimethylammonium bromide (CTAB) to quantify enzymatic activity.

In each well, the following components were combined: 10 μL of the test extracts (0.01 to 10 mg/mL in 0.1M acetate buffer, pH 5.35), 15 μL of sodium acetate buffer (0.1M acetate buffer, pH 5.35), 25 μL of incubation buffer (acetate buffer, pH 5.35; containing 0.1 mg/mL bovine serum albumin [BSA] and 4.5 mg/mL sodium chloride), and 25 μL of bovine hyaluronidase enzyme solution (30 U/mL, prepared in incubation buffer). The mixture was incubated at 37 °C for 10 min. Subsequently, 25 μL of hyaluronic acid solution (0.3 mg/mL in acetate buffer, pH 5.35) was added to each well, followed by a second incubation period of 45 min at 37 °C.

After enzymatic digestion, the undigested HA was precipitated by adding 200 μL of CTAB (2.5% in distilled water) to each well. Plates were then kept at 25 °C for 10 min to allow for complex formation. The turbidity resulting from HA–CTAB complexation was measured spectrophotometrically at 600 nm.

$${INH}_{hyal}=\left(\frac{A_{S}-A_{C}}{A_{T}-A_{C}}\right)*100\%$$

A_S_—absorbance of the substrate + sample + enzyme;

A_C_—absorbance of the substrate + enzyme;

A_T_—absorbance of the substrate + sample.

α-Amylase Inhibitory Assay

Amylase activity was determined using a colorimetric assay based on the formation of a blue complex between iodine and residual, non-hydrolyzed starch [18]. Briefly, 25 μL of the tested extract (0.01 to 10 mg/mL in 20 mM PBS, pH 7.6) or the reference inhibitor (acarbose) was combined with 50 μL of α-amylase solution (10 U/mL, from porcine pancreas, prepared in 20 mM PBS, pH 7.6) and 50 μL of starch solution (0.05% in distilled water). The reaction mixture was incubated at 37 °C for 10 min. The reaction was then terminated by adding 25 μL of 1 M hydrochloric acid, followed by the addition of 100 μL of 3% iodine solution (in ethanol). Absorbance was recorded at 600 nm using a spectrophotometer. The percentage of enzyme inhibition was calculated based on the difference in absorbance between the control and the tested samples according to the appropriate formula:

$${INH}_{Amylase}=(1-\frac{A_{C}-A_{S}}{A_{C}})*100\%$$

A_S_—absorbance of the substrate + sample + enzyme;

A_C_—absorbance of the substrate + enzyme.

Lipase inhibition assay

Pancreatic lipase inhibitory activity was assessed using p-nitrophenyl dodecanoate (*p*-NPD, 10 mM) as the substrate and porcine pancreatic lipase (1 mg/mL) prepared in Tris-HCl buffer (100 mM, pH 8.0) [19]. The plant extracts were tested at concentrations ranging from 0.01 to 10 mg/mL, and orlistat was used as a positive control.

For the assay, 25 μL of the tested extract (0.01 to 10 mg/mL in Tris-HCl buffer, 100 mM, pH 8.0) was combined with 50 μL of enzyme solution (1 mg/mL in Tris-HCl buffer, 100 mM, pH 8.0) and pre-incubated at 37 °C for 10 min. Next, 10 μL of *p*-NPD solution (5 mM in isopropanol) was added, and the total reaction volume was adjusted to 200 μL with Tris-HCl buffer. The mixtures were incubated at 37 °C, and absorbance was measured at 405 nm after 5, 10, 15, and 20 min using a microplate reader to confirm the linearity of product formation. The inhibition values were calculated using absorbance data obtained within the linear phase of the reaction.

Orlistat was used as the positive control inhibitor. The percentage of enzyme inhibition was calculated using the following formula:

$${INH}_{lipase}=(1-\frac{{\Delta A}_{C}-{\Delta A}_{S}}{{\Delta A}_{C}})*100\%$$

A_S_—the difference in absorbance between time t_2_ and t_1_ for the sample;

A_C_—the difference in absorbance between time t_2_ and t_1_ for the positive control.

Tyrosinase Inhibitory Assay

The inhibitory activity against tyrosinase was determined using a microplate-based assay performed in 96-well plates, following a slightly modified procedure based on the method reported by Gębalski et al. [20]. Briefly, 10 μL of the tested sample (0.01 to 10 mg/mL in 50 mM phosphate buffer, pH 6.8) was mixed with 140 μL of phosphate buffer (50 mM, pH 6.8) and 25 μL of tyrosinase enzyme solution (125 U/ML tyrosinase from mushroom, prepared in 50 mM phosphate buffer, pH 6.8). The reaction mixture was incubated at room temperature for 10 min. A control sample without inhibitor (Ac) was prepared under the same conditions. After the incubation period, 25 μL of L-tyrosine solution (0.3 mg/mL in phosphate buffer 50 mM, pH 6.8) was added to initiate the enzymatic reaction. The formation of dopachrome was monitored spectrophotometrically at 510 nm using a kinetic measurement mode with readings taken every 5 min. Two time points (t_1_ and t_2_) within the linear portion of the reaction curve were selected for calculations. Each sample was analyzed in triplicate. Kojic acid was used as the reference inhibitor. The percentage of tyrosinase inhibition was calculated according to the following equation:

$${INH}_{tyrosinase}=(\frac{\Delta A_{C}-{\Delta A}_{S}}{{\Delta A}_{C}})*100\%$$

A_S_—the difference in absorbance between time t_2_ and t_1_ for the sample;

A_C_—the difference in absorbance between time t_2_ and t_1_ for the positive control.

Butyrylcholinesterase Inhibitory Assay

The inhibitory activity toward butyrylcholinesterase (BuChE) was evaluated using a spectrophotometric method based on Ellman’s reaction with slight modifications [21,22]. Briefly, 5 μL of the tested extract (0.01 to 10 mg/mL) was combined with 45 μL of enzyme solution (0.4 U in phosphate buffer 50 mM, pH 7.4) and pre-incubated for 15 min at room temperature. Following incubation, 150 μL of the reaction mixture containing phosphate buffer (50 mM, pH 7.4), acetylcholine substrate (0.1 M in distilled water), and 5,5′-dithiobis-(2-nitrobenzoic acid) (10 mM in methanol) in a ratio of 308:2:1 (*v*/*v*/*v*) was added.

The enzymatic reaction was monitored spectrophotometrically at 405 nm immediately after the addition of the substrate and again after 10 min. Donepezil served as the positive control inhibitor. The percentage inhibition of enzyme activity was calculated according to the following equation:

$${INH}_{BuChE}=\left(1-\frac{As}{Ac}\right)*100\%$$

A_S_—absorbance of the substrate + sample + enzyme;

A_C_—absorbance of the substrate + enzyme.

#### 2.5.2. Antioxidant Assays

All test samples were dissolved in buffer prior to analysis. The extracts were evaluated at concentrations ranging from 0.01 to 10 mg/mL. The IC_50_ values were determined by fitting the dose–response curves using nonlinear regression analysis. The IC_50_ value was defined as the concentration required to achieve 50% of the maximal radical scavenging activity.

DPPH assay

Antioxidant activity was assessed using a 96-well microplate format [23,24]. The working 2,2-diphenyl-1-picrylhydrazyl (DPPH) solution was prepared by dissolving 24 mg of DPPH in 100 mL of methanol, and its absorbance was adjusted to 0.900 ± 0.03 at 515 nm. In each well, 10 μL of the test extracts (0.01 to 10 mg/mL) was mixed with 190 μL of the DPPH solution. The background control consisted of 10 μL of PBS (50 mM, pH 7.4) and 190 μL of the DPPH solution. Trolox was used as the reference antioxidant. The plate was incubated in the dark for 60 min, and absorbance was measured at 515 nm. The DPPH radical scavenging activity was expressed as the percentage of inhibition, calculated using the following equation.

$${INH}_{DPPH}=(\frac{A_{C}-A_{S}}{A_{C}})*100\%$$

A_S_—absorbance of the sample + DPPH;

A_C_—absorbance of the DPPH.

ABTS assay

The ABTS radical scavenging assay was carried out using a 96-well microplate format [24,25]. The 2,2′-azinobis(3-ethylbenzothiazoline-6-sulfonic acid) radical cation (ABTS^•+^) stock solution was prepared by combining equal volumes of 7 mM 2,2′-azinobis(3-ethylbenzothiazoline-6-sulfonic acid) (ABTS) aqueous solution and 2.45 mM potassium persulfate solution (10 mL each). The resulting mixture was incubated in the dark for 12 h to allow for the generation of ABTS radicals. Following incubation, the solution was diluted with distilled water until an absorbance of 0.700 ± 0.03 was obtained at 405 nm. In each well, 10 μL of the test extracts (0.01 to 10 mg/mL) was added to 190 μL of the prepared ABTS solution. As a control, 10 μL of buffer PBS (50 mM, pH 7.4) was combined with 190 μL of the ABTS solution. The reference antioxidant used in the assay included Trolox. The plate was then incubated in the dark for 30 min prior to measuring the absorbance at 405 nm. The percentage inhibition of ABTS radicals was calculated using the following formula:

$${INH}_{ABTS}=(\frac{A_{C}-A_{S}}{A_{C}})*100\%$$

A_S_—absorbance of the sample + ABTS;

A_C_—absorbance of the ABTS.

Iron (II) Ion Chelation Assay

The assay was performed using a 96-well microplate format [26]. Each test sample was prepared at a final concentration of 1 mg/mL (50 mM PBS, pH 7.4). For each well, 140 μL of methanol, 5 μL of FeCl_2_ solution (2 mM in distilled water), and 100 μL of the test extracts were added. Control wells contained 140 μL of methanol, 5 μL of FeCl_2_ solution, and 100 μL of buffer. The plate was incubated at 25 °C for 5 min, followed by the addition of 5 μL of ferrozine solution (5 mM in distilled water) to each well. After an additional 10-min incubation at 25 °C, absorbance was recorded at 517 nm. The percentage of metal chelating activity was calculated using the following equation:

$${INH}_{Chel.}=(\frac{A_{C}-A_{S}}{A_{C}})*100\%$$

A_S_—absorbance of the sample + Ferrozine + Fe^2+^;

A_C_—absorbance of the Ferrozine + Fe^2+^.

Ferric Ion Reducing Antioxidant Power Assay

The ferric reducing antioxidant power (FRAP) assay was carried out by mixing the test extract, prepared at a concentration of 1.0 mg/mL (300 mM acetate buffer, pH 3.6), with 290 μL of the FRAP working solution. This solution was composed of 15 mL of acetate buffer (300 mM, pH 3.6), 1.5 mL of TPTZ reagent (10 mM in 40 mM HCl), and 1.5 mL of FeCl_3_·4H_2_O solution (20 mM in distilled water). The reaction mixture was incubated for 30 min at room temperature, after which the absorbance was recorded at 593 nm. Trolox and butylated hydroxyanisole (BHA) were included as standard antioxidants. The antioxidant capacity was expressed as micrograms of Trolox equivalent per gram of dry extract (µg Trolox/g DE) [27].

Cupric Ion Reducing Antioxidant Capacity Assay

The cupric ion reducing antioxidant capacity (CUPRAC) assay was conducted by adding 10 μL of each extract, prepared at a concentration of 1.0 mg/mL (1 M acetate buffer, pH = 7), to 190 μL of the CUPRAC reagent mixture. This working solution consisted of acetate buffer (1M acetate buffer, pH 7.0), neocuproine (7.5 mM in methanol), and copper (II) chloride (10 mM in distilled water), mixed in equal volumes (1:1:1, *v*/*v*/*v*). The reaction mixture was incubated at room temperature for 15 min, after which absorbance was measured at 450 nm. Antioxidant capacity was expressed as micrograms of Trolox equivalents per gram of dry extract (µg Trolox/g DE) [28].

Superoxide Anion Radical Scavenging Capacity Assay

Superoxide anion radical scavenging activity was evaluated using the xanthine–xanthine oxidase system in the presence of nitro blue tetrazolium chloride (NBT), following the method reported by Choi et al. [29], with minor modifications. In brief, 50 μL of each extract solution (0.01–10 mg/mL) was mixed with 100 μL of a reaction mixture containing xanthine (0.4 mM) and NBT (0.24 mM), both prepared in 50 mM phosphate-buffered saline (PBS, pH 7.4), in a 1:1 (*v*/*v*) ratio. Subsequently, 50 μL of xanthine oxidase (10 mU in 50 mM PBS, pH 7.4) was added to initiate the reaction. The mixture was incubated at 37 °C for 20 min, after which absorbance was measured at 560 nm. Ascorbic acid served as the reference antioxidant.

$${INH}_{O_{2}^{•-}}=(\frac{A_{C}-A_{S}}{A_{C}})*100\%$$

A_S_—absorbance of the sample + NBT + xanthine–xanthine oxidase system;

A_C_—absorbance of the NBT + xanthine–xanthine oxidase system.

#### 2.5.3. Lipid Peroxidation Assays

Animals

Specimens were obtained from one adult albino New Zealand rabbit, weighing 3.5–4.0 kg and aged 3–4 months. The animal was maintained under a 12 h light/12 h dark cycle with free access to food and water. Euthanasia was performed by exposure to a high concentration of isoflurane followed by carbon dioxide inhalation (approximately 60% of the inhaled air). All experimental procedures were conducted in accordance with the European Directive 2010/63/EU for animal experiments. The study protocol was approved by the Local Ethics Committee for Animal Experiments at the Ethical Committee in Bydgoszcz, 11 October 2016 (approval no. 16/2016).

Preparation of Rabbit Plasma

Following euthanasia, blood samples were collected into tubes containing high-molecular-weight heparin as an anticoagulant and kept on ice until processing. Plasma was obtained by centrifugation at 3000× *g* for 10 min at 4 °C. The collected plasma was immediately separated, aliquoted, and stored at −80 °C until further antioxidant analyses.

AAPH-Induced TBARS Method

Lipid peroxidation was assessed using the thiobarbituric acid reactive substances (TBARS) assay following oxidative stress induction with 2,2′-azobis(2-amidinopropane) dihydrochloride (AAPH). The reaction mixture (final volume 1.0 mL) contained:

100 µL of fatty acid or plasma sample;

100 µL of the tested extract (phosphate buffer, 0.1 M, pH 7.4);

100 µL of AAPH solution (10 mM in phosphate buffer, 0.1 M, pH 7.4);

700 µL of phosphate buffer (0.1 M, pH 7.4).

Samples were incubated for 4 h at 37 °C to allow generation of peroxyl radicals and induction of lipid peroxidation. After incubation, 1 mL of an aqueous solution containing 0.67% (*w*/*v*) TBA and 10% (*w*/*v*) TCA, both dissolved in distilled water, was added. The samples were heated in boiling water for 30 min to allow formation of the MDA–TBA adduct. Subsequently, the samples were cooled on ice for 10 min and centrifuged to remove precipitated material. Aliquots of 200 µL of the supernatant were transferred in triplicate to a 96-well microplate, and absorbance was measured at 532 nm using a microplate reader.

For assays performed with linoleic acid, the results were expressed as nmol MDA/mL. For plasma samples, the level of lipid peroxidation was expressed as nmol malondialdehyde per mg of protein (nmol MDA/mg protein) [30]. Protein concentration was determined using the Bradford method with bovine serum albumin (BSA) as a standard [31].

### 2.6. Statistical Analysis

Statistical analysis was performed using GraphPad Prism (version 10.0, GraphPad Software, San Diego, CA, USA), Microsoft Excel (Microsoft Corporation, Redmond, WA, USA), and Statistica software (version 13.3, StatSoft, Tulsa, OK, USA). All experiments were conducted in triplicate, and the results are presented as mean ± standard deviation (SD). Statistical differences between samples were evaluated using one-way analysis of variance (ANOVA) followed by Tukey’s post hoc test. Differences were considered statistically significant at *p* < 0.05. Different superscript lowercase letters indicate statistically significant differences within the same column. IC_50_ values for DPPH and ABTS assays were determined using nonlinear regression analysis based on dose–response curves, whereas IC_50_ values for enzymatic assays were calculated using linear regression analysis.

## 3. Results and Discussion

### 3.1. Phytochemical Profiling

Phytochemical analysis demonstrated clear qualitative and quantitative differences among the examined species and between roots and aerial parts. Significant differences in total phenolic content were observed among the investigated samples (Table 1). The highest TPC was detected in the root of AM (199.84 ± 3.64 mg GAE/g DE), whereas the remaining samples contained lower amounts, ranging from 127.25 ± 1.99 to 150.98 ± 1.75 mg GAE/g DE. In contrast, the aerial parts of AC and AG showed comparable phenolic levels, reaching 150.98 ± 1.75 and 129.77 ± 2.23 mg GAE/g DE, respectively.

The distribution of flavonoids differed substantially among the investigated species and plant organs (Table 1). The highest TFC was detected in the aerial part of AC (44.64 ± 2.91 mg QE/g DE), closely followed by the root of AM (43.23 ± 1.58 mg QE/g DE). In contrast, the lowest flavonoid levels were found in the roots of AG and AC (22.47 ± 4.49 and 27.04 ± 3.00 mg QE/g DE, respectively), while the remaining extracts showed intermediate values.

Total phenolic acids were detected exclusively in the aerial parts of AM and AC. The highest total phenolic acid content (TPAC) was observed in the herb of AM (11.38 ± 0.72 mg CAE/g DE), whereas the herb of AC contained substantially lower amounts (2.70 ± 0.31 mg CAE/g DE). No phenolic acids were detected in the roots of any investigated species or in the herb of AG.

The results indicate significant interspecies and organ-dependent variability in phenolic composition. Roots, particularly those of AM, were distinguished by the highest overall phenolic concentration, whereas aerial parts represented the primary source of flavonoids and phenolic acids. These compositional differences likely contribute to the distinct biological activity profiles observed among the examined extracts. The results are shown in Table 1.

Similar organ-dependent trends were described by Shahrivari-Baviloliaei et al. for AC and AG. In that study, hydromethanolic extracts were prepared using 75% methanol assisted by ultrasound extraction. Total phenolic content ranged from 0.88 to 8.30 µg GAE/g Dry Weight (DW), with the highest value recorded in AC leaves (8.30 ± 0.24 µg GAE/g DW), whereas roots contained substantially lower amounts (0.88 ± 0.67 µg GAE/g DW in AG and 3.67 ± 0.91 µg GAE/g DW in AC). A similar distribution was observed for total flavonoids, reaching 3.27 ± 0.65 µg QE/g DW in AC leaves and 2.43 ± 0.22 µg QE/g DW in AG leaves, while roots contained only 0.12–0.21 µg QE/g DW. In contrast, aqueous extracts prepared as infusions contained considerably higher amounts of phenolic compounds. Total phenolic content reached 78.29 ± 2.03 µg GAE/g DW in AC leaves and 70.83 ± 1.82 µg GAE/g DW in AG leaves, whereas total flavonoid content exceeded 1100 µg QE/g DW in leaf samples [32].

In a previous study, the phytochemical composition of commercially obtained AM root was evaluated using 75% methanolic extracts prepared by ultrasound-assisted extraction. The results, expressed per gram of dry extract, showed that TPC reached 87.90 ± 6.47 mg GAE/g DE, while TFC amounted to 20.90 ± 0.67 mg QE/g DE. In contrast, TPAC was considerably lower, reaching 1.11 ± 0.00 mg CAE/g DE. These findings indicate that phenolic compounds constitute an important fraction of the phytochemical profile of AM root extracts and may contribute to their biological activity [24].

Similarly, Shahrivari-Baviloliaei et al. investigated commercial samples of AM using both hydromethanolic extracts obtained by ultrasound-assisted extraction with methanol–water (80:20, *v*/*v*) and aqueous extracts prepared as infusions. In hydromethanolic extracts, total phenolic content ranged from 325.31 to 577.70 µg GAE/g DW, while total flavonoid content varied between 59.32 and 170.39 µg QE/g DW. In contrast, water extracts contained higher levels, with TPC reaching up to 3360 µg GAE/g DW and TFC up to 1396.14 µg QE/g DW in the richest sample [33].

The observed differences among studies may be attributed not only to species- and organ-specific variation but also to differences in extraction procedures and solvent systems. Extraction efficiency strongly depends on solvent polarity and the physicochemical properties of the target compounds. Highly polar compounds, including many phenolic acids and flavonoid glycosides, are often extracted more efficiently with water, whereas hydroalcoholic mixtures enable the simultaneous extraction of compounds with a broader range of polarities.

The UHPLC–DAD–ESI/TOF–MS profile demonstrated the predominance of different groups of compounds depending on the species and plant part. Example chromatograms are shown in Figures S1–S6. Mass data summarized in Tables S1–S6 were used to generate chemical formulas, while UV–Vis spectra were used to confirm the compound classes. Flavonoids exhibited characteristic absorption maxima at ~265 and 365 nm for kaempferol derivatives, ~255 and 370 nm for isorhamnetin derivatives, and ~255–265 and 370–375 nm for quercetin derivatives. Derivatives of caffeic, chlorogenic, and ferulic acids showed absorption maxima at ~320–330 nm. Due to the lack of standards for phenolic acid derivatives and high-molecular-weight flavonoids, fragmentation patterns were further used to establish the parent aglycone. Selected representative UV–Vis and MS spectra are included in the Supplementary Materials (Figure S7). Detailed quantitative data are provided in the Supplementary Materials (Tables S1–S6), while validation parameters, including calibration curves, linear equations, and correlation coefficients, are summarized in Table S7. Among polyphenolic compounds in the herb of AM, kaempferol derivatives were the major constituents. One compound was present at the highest concentration (63.44 ± 0.69 mg/g of dried extract; *m*/*z* 739.207 [M−H]^−^; molecular formula C_33_H_40_O_19_). Other kaempferol derivatives were detected at 3.94 ± 0.12 mg/g, 2.96 ± 0.11 mg/g, and 1.92 ± 0.10 mg/g (*m*/*z* 871.254 [M−H]^−^: C_38_H_48_O_23_; *m*/*z* 593.150 [M−H]^−^: C_27_H_30_O_15_; and *m*/*z* 901.260 [M−H]^−^: C_39_H_50_O_24_, respectively). Their assignment to kaempferol derivatives was confirmed based on the similarity of UV–Vis spectra and the presence of a characteristic fragment ion at *m*/*z* 284; however, it should be emphasized that further studies are required to confirm the structures of these compounds due to the limited availability of specific literature. The presence of high-molecular-weight flavonoid compounds in *Astragalus* species was also observed by other researchers [34,35]. In the root of this species, dicaffeoyltartaric acid predominated (108.6 ± 5.15 mg/g DE), followed by caftaric acids and their hexosides (total: 9.90 ± 0.43 mg/g DE). Minor amounts of other phenolic acids were also observed, including feruloyltartaric acid, caffeoyl-feruloyltartaric acid, and caffeoyl glucose (Table S2).

The herb of AC was characterized by a high content of flavonoids, including a compound with *m*/*z* 593.1507 [M−H]^−^ (estimated molecular formula C_27_H_30_O_15_) at 19.96 ± 0.89 mg/g DE, as well as a vitexin derivative (8.26 ± 0.27 mg/g DE). Various kaempferol derivatives were also detected, including alangiflavoside (6.66 ± 0.31 mg/g) and compounds with *m*/*z* 1077.31071 [M−H]^−^ (C_49_H_58_O_27_; total: 6.38 mg/g DE). Smaller amounts of other flavonoid derivatives were also observed (Table S3). Esters of caffeic and ferulic acids with glucaric acid were also present. The root of AC was poor in phenolic compounds. Identified constituents included isovanillic acid, glucosyringic acid, and *p*-coumaroyltartaric acid (total ≈ 1.0 mg/g DE), as well as kaempferol hexosides (0.16 mg/g). Similarly, the root of AG contained only small amounts of phenolic compounds, including derivatives of gentisic acid, glucosyringic acid, and hydroxybenzoic acid. In contrast, the herb of AG consisted of relatively high amounts of hydroxycinnamic acid derivatives, particularly *p*-coumaroylquinic and feruloylquinic acids, which occurred as multiple positional isomers. Among them, 4-*p*-coumaroylquinic acid derivatives reached the highest levels (ca. 5.0 mg/g DE), followed by the 3- and 5-isomers (ca. 3.0 mg/g DE and 0.78 mg/g DE, respectively), while feruloylquinic acid derivatives were present in lower amounts (total ca. 2.44 mg/g DE), with 4-feruloylquinic acid being predominant (ca. 1.0 mg/g DE). Flavonoids were mainly represented by numerous kaempferol derivatives. The most abundant compounds in this group included derivatives with *m*/*z* 741.19636 [M−H]^−^ (C_32_H_38_O_20_; 3.14 ± 0.12 mg/g DE), *m*/*z* 755.20539 [M−H]^−^ (C_33_H_40_O_20_; 2.66 ± 0.11 mg/g DE), and *m*/*z* 887.25007 [M−H]^−^ (C_38_H_48_O_24_; 1.84 mg/g DE), as well as kaempferol dihexoside (1.39 ± 0.05 mg/g DE). The results indicate that the aerial parts of all examined species are a richer source of flavonoids, whereas the root of AM is distinguished by the highest total phenolic content, which may directly contribute to the observed biological activity of the extracts. Table 2 presents quantitative data as sums of derivatives of the respective aglycones.

Followed by a literature report on the presence of isoflavones in *Astragalus* species [36,37], the presence of these compounds was also verified using Extracted Ion Chromatogram (EIC) and comparison with reference standards. Small amounts of formononetin, calycosin, and their glycosylated derivatives were detected, with the highest content found in the root of AM (total 339.81 ± 10.61 µg/g DE), followed by the root of AG (327.54 ± 12.05 µg/g DE) (Table S8). Furthermore, in positive ionization, cycloartane-type saponins with a characteristic mass ion at *m*/*z* 455 ([aglycone + H − H_2_O]^+^) [38] were also observed in all extracts (Figure S8). These compounds are considered markers of *Astragalus* species [36,37,38,39]. Although our analysis did not confirm the presence of astragalosides I–IV reported in the literature, this may be attributed to the structural complexity and diversity of these compounds. Based on signal abundance, the extract from the root of AG appeared to be particularly rich in compounds of this type.

HPLC analysis reported by other researchers revealed qualitative and quantitative differences in the phenolic profiles of AC and AG depending on plant part and extraction solvent. In methanolic extracts, gallic acid and quercetin were the predominant compounds. In AG leaves, gallic acid reached 89.93 ± 0.84 µg/g DW and quercetin 336.27 ± 6.24 µg/g DW, whereas roots contained 75.82 ± 1.33 µg/g DW and 35.62 ± 1.76 µg/g DW, respectively. In fruits, rutin was detected at 72.17 ± 1.37 µg/g DW and quercetin at 48.00 ± 2.40 µg/g DW. In AC, methanolic extracts of leaves contained 49.84 ± 6.55 µg/g DW of gallic acid and 82.78 ± 2.11 µg/g DW of quercetin, while roots and fruits were characterized mainly by quercetin, reaching 37.27 ± 3.46 µg/g DW and 51.15 ± 5.18 µg/g DW, respectively. Other phenolic acids, such as ferulic acid and cinnamic acid, were detected selectively, primarily in AG leaves and fruits. Water extracts exhibited higher concentrations of gallic acid, reaching 674.50 ± 12.22 µg/g DW in AG fruits and 435.49 ± 13.75 µg/g DW in AC leaves, while quercetin ranged from 43.71 ± 0.12 µg/g DW in AG to 75.60 ± 1.02 µg/g DW in AC fruits [32].

In the study by Shahrivari-Baviloliaei et al., the phenolic composition of hydro-methanolic and water extracts of commercial AM root samples was investigated. Ten phenolic compounds were analyzed using HPLC, and quercetin and gallic acid were identified as the predominant constituents. In hydro-methanolic extracts, quercetin reached concentrations up to 228.34 µg/g DW, while rutin was also detected at relatively high levels, up to 140.74 µg/g DW. In contrast, water extracts contained mainly gallic acid and quercetin, with gallic acid ranging from 315.34 to 481.40 µg/g DW and quercetin from 56.88 to 163.95 µg/g DW depending on the analyzed sample [33]. In turn, in the study by Santoro et al., the chemical composition of the 50% ethanol of AM root extracts was investigated using LC–MS/MS. The analysis allowed the identification of 31 compounds, including 19 polyphenols and 12 triterpenoid saponins. Among the most important polyphenolic constituents were isoflavonoids such as calycosin, calycosin-7-*O*-glucoside, formononetin and daidzein [40].

The saponin fraction was mainly represented by cycloartane-type glycosides, including astragalosides I–IV as well as soyasaponin I, which are considered key bioactive compounds and important markers of AM roots [41].

### 3.2. Enzyme Inhibitory Activity

The strongest hyaluronidase inhibition was observed for the root of AM (IC_50_ = 214.51 ± 52.75 µg/mL), showing activity comparable to escin. The remaining extracts exhibited weaker inhibitory effects, with IC_50_ values exceeding 400 µg/mL. The results are shown in Table 3.

In one study investigating the anti-enzymatic activity of adaptogenic plants, methanolic extracts of AM root showed moderate inhibitory activity against hyaluronidase. The inhibition was concentration-dependent and reached approximately 60% at a concentration of 10 mg/mL, while at 1 mg/mL the activity decreased to about 10%. These results indicate that relatively high concentrations of the extract are required to obtain noticeable inhibition of this enzyme [24]. The observed differences between roots and aerial parts may reflect variations in phenolic composition, particularly the higher total phenolic content detected in AMR.

Tyrosinase inhibitory activity was observed only for the root extract of AM, which demonstrated moderate activity (IC_50_ = 37.32 ± 0.35 µg/mL), while the remaining extracts were inactive under the applied experimental conditions. Among the tested standards, calycosin exhibited strong inhibitory activity (IC_50_ = 3.4 ± 1.2 µg/mL), comparable to that of the reference inhibitor kojic acid (IC_50_ = 2.93 ± 0.17 µg/mL). In contrast, calycosin-7-O-*β*-D-glucoside showed substantially weaker activity (IC_50_ = 379.8 ± 11.23 µg/mL), suggesting that glycosylation markedly reduces tyrosinase inhibitory potential. Kim et al. isolated calycosin-7-O-*β*-D-glucoside from a methanolic extract of AM and evaluated its inhibitory activity against tyrosinase. The compound showed dose-dependent inhibition of mushroom tyrosinase, with an IC_50_ value of 68 µM. This activity was comparable to that of the reference inhibitor kojic acid, which exhibited an IC_50_ value of 79.5 µM under the same experimental conditions [42].

The root extract of AM exhibited the strongest α-amylase inhibitory activity among all investigated samples (IC_50_ = 17.78 ± 1.16 µg/mL), although it was less potent than the reference inhibitor acarbose (IC_50_ = 0.56 ± 0.03 µg/mL). Considerably weaker activity was observed for the herb of AM and the root of AC, whereas the remaining extracts showed little or no inhibitory effect. Among the tested standards, formononetin demonstrated notable activity (IC_50_ = 4.95 µg/mL), while its glycosylated derivative exhibited lower potency, suggesting that glycosylation may reduce α-amylase inhibitory activity. Similar observations indicating the ability of *Astragalus* species to inhibit carbohydrate-digesting enzymes have been reported previously. In *A. kurdicus* Boiss., root extracts showed strong inhibitory activity at 1 mg/mL, with the water extract being the most effective (91.49 ± 0.06%), followed by methanolic, chloroform, and butanol. Notably, its activity exceeded that of acarbose (88.89 ± 0.01%), suggesting an important role of polar constituents in enzyme inhibition [43]. In the study by Güven et al., methanolic and water extracts of *A. alopecurus* inhibited α-amylase, with stronger activity observed for the aqueous extract (IC_50_ = 346.58 µg/mL) than the methanolic one (IC_50_ = 693.15 µg/mL). However, acarbose showed much higher potency (IC_50_ = 6.46 µg/mL), suggesting that polar constituents contribute to enzyme inhibition [44].

Pancreatic lipase inhibitory activity was observed only for the root extracts of AM and AC, with the strongest effect recorded for AM (IC_50_ = 226.7 ± 18.0 µg/mL). The remaining extracts did not exhibit measurable activity under the applied experimental conditions. Among the tested standards, astragaloside IV showed the highest inhibitory activity (IC_50_ = 150.70 ± 11.30 µg/mL), followed by astragaloside III (IC_50_ = 292.80 ± 29.9 µg/mL), suggesting that triterpenoid saponins may contribute to the lipase inhibitory potential of *Astragalus* extracts. However, all tested samples were markedly less active than the reference inhibitor orlistat (IC_50_ = 1.17 ± 0.04 µg/mL). Ben Khaled et al. investigated the lipase inhibitory activity of a polysaccharide extract obtained from the leaves of *A. spinosus* (Forssk.) Muschl. The study demonstrated strong, concentration-dependent inhibition, with activity increasing between 1.5 and 2.0 mg/mL and reaching complete inhibition at 2.5–3.0 mg/mL [45]. The results indicate that lipase inhibition by *Astragalus* extracts is moderate and limited mainly to root-derived samples.

No inhibitory activity against BuChE was observed for any of the tested *Astragalus* extracts under the applied experimental conditions. In contrast, the reference inhibitor donepezil demonstrated clear activity, with an IC_50_ value of 6.79 ± 0.51 µg/mL.

### 3.3. In Vitro Antioxidant Activity

The antioxidant activity assays demonstrated differences between species and plant parts, which were consistent with the previously observed variations in polyphenolic composition. In the DPPH radical scavenging assay, the strongest activity was observed for the root of AM, with an IC_50_ value of 36.53 ± 1.22 µg/mL. The herb of AC showed moderate activity, IC_50_ = 136.31 ± 2.03 µg/mL, whereas significantly weaker effects were recorded for ACR (404.80 ± 120.15 µg/mL), AGR (559.70 ± 48.84 µg/mL), AGH (603.36 ± 7.35 µg/mL), and AMH (615.99 ± 29.98 µg/mL). The reference compound BHA exhibited an IC_50_ of 21.49 ± 0.29 µg/mL. In the ABTS assay, AMR again demonstrated the highest radical scavenging capacity, IC_50_ = 26.31 ± 0.03 µg/mL, comparable to the activity of BHA (26.31 ± 0.03 µg/mL). The herb of AC also showed relatively strong activity, IC_50_ = 43.18 ± 0.74 µg/mL. The remaining extracts displayed weaker effects, with IC_50_ values ranging from 126.47 ± 5.20 µg/mL (AGR) to 151.84 ± 2.79 µg/mL (AMH). The ferrous ion chelating assay revealed a different activity pattern. The strongest chelating properties were observed for AGH, IC_50_ = 8.57 ± 1.37 µg/mL, followed by ACR (17.80 ± 2.66 µg/mL) and ACH (18.90 ± 1.74 µg/mL). AMR showed moderate activity, IC_50_ = 42.64 ± 1.14 µg/mL, while AMH and AGR were less effective, with IC_50_ values of 58.68 ± 1.37 µg/mL and 54.29 ± 5.04 µg/mL, respectively. As expected, EDTA exhibited nearly complete chelation (100 ± 0.01%). In the CUPRAC assay, the highest reducing capacity was again observed for AMR, reaching 24.43 ± 0.62 µg Trolox/g DE. ACH also demonstrated notable activity, 8.01 ± 0.55 µg Trolox/g DE, whereas the remaining extracts showed considerably lower values, ranging from 1.31 ± 0.14 µg Trolox/g DE (AGR) to 1.82 ± 0.29 µg Trolox/g DE (ACR). Similarly, in the FRAP assay, AMR exhibited the strongest ferric reducing power, 3.37 ± 0.23 µg Trolox/g DE. ACH followed with 1.82 ± 0.18 µg Trolox/g DE, while the other samples showed substantially lower reducing capacity, between 0.29 ± 0.03 µg Trolox/g DE (AGH) and 0.50 ± 0.09 µg Trolox/g DE (ACR).

The superoxide anion radical scavenging activity varied among the investigated extracts. The strongest activity was observed for ACH (IC_50_ = 114.6 ± 15.0 µg/mL). The remaining extracts exhibited weaker activity. Ascorbic acid, used as a reference antioxidant, showed significantly stronger activity, with an IC_50_ value of 35.55 ± 0.22 µg/mL. The results are presented in Table 4.

The antioxidant activity of AC and AG methanolic extracts was evaluated using DPPH, FRAP, and CUPRAC assays. In AG, DPPH scavenging activity reached 4.54 ± 0.18 mg TE/g DW in leaves, compared with 1.99 ± 0.72 mg TE/g DW in roots. Correspondingly, FRAP values were 130.60 ± 8.38 mg Fe^2+^/g DW in leaves and 47.77 ± 5.51 mg Fe^2+^/g DW in roots, while CUPRAC activity amounted to 34.77 ± 1.99 and 3.43 ± 0.62 mg AA/g DW, respectively. AC extracts were generally characterized by higher reducing power. In hydro-methanolic leaf extracts, FRAP and CUPRAC values were higher than in AG, confirming stronger electron-donating capacity [32].

In contrast, commercial AM samples demonstrated substantially higher antioxidant parameters. Methanolic extracts showed DPPH values ranging from 6.31 to 13.29 mg TE/g DW and FRAP values from 52.43 to 99.74 mg Fe^2+^/g DW, while aqueous extracts exhibited even stronger activity, with DPPH up to 25.87 mg TE/g DW and FRAP up to 165.32 mg Fe^2+^/g DW. Notably, strong positive correlations between total phenolic content and reducing capacity, particularly for FRAP and CUPRAC assays, confirmed the dominant role of phenolic constituents in determining antioxidant potential [24].

### 3.4. Lipid Peroxidation Assays

#### 3.4.1. Linoleic Acid Oxidation Assay

**Figure 1:**
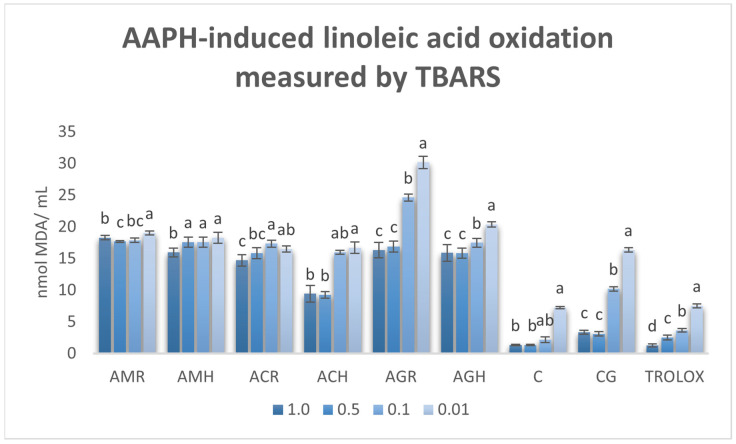
Effect of *Astragalus* extracts on AAPH-induced lipid peroxidation in the linoleic acid model system. Lipid peroxidation was determined using the TBARS method and expressed as malondialdehyde (MDA) equivalents. AMR—*Astragalus membranaceus* root, AMH—*Astragalus membranaceus* herb, ACR—*Astragalus cicer* root, ACH—*Astragalus cicer* herb, AGR—*Astragalus glycyphyllos* root, AGH—*Astragalus glycyphyllos* herb, CG—Calycosin-7-O-*β*-D-glucoside, C—Calycosin. Different superscript lowercase letters indicate a statistically significant difference between the fractions themselves and between the fractions and control within the same column, with *p* < 0.05.

The effects of *Astragalus* extracts on AAPH-induced lipid peroxidation in the linoleic acid model are presented in Figure 1. Among the tested extracts, the herb of AC exhibited the strongest protective effect, with the lowest MDA levels observed at 0.1 and 0.05 mg/mL (9.42 and 9.22 nmol MDA/mL, respectively). Moderate inhibition of lipid peroxidation was recorded for extracts from AM, whereas the root of AG showed the weakest activity, particularly at lower concentrations, where MDA levels increased markedly. The reference compounds demonstrated substantially stronger antioxidant effects than the plant extracts. Calycosin was the most active standard, reducing MDA formation to 1.32 nmol MDA/mL at the highest tested concentrations, while Trolox showed the strongest overall protective effect across the entire concentration range. These findings indicate that although *Astragalus* extracts can attenuate lipid peroxidation, their activity is considerably lower than that of pure antioxidant standards.

#### 3.4.2. Effect of Astragalus Extracts on AAPH-Induced Lipid Peroxidation in Plasma

**Figure 2:**
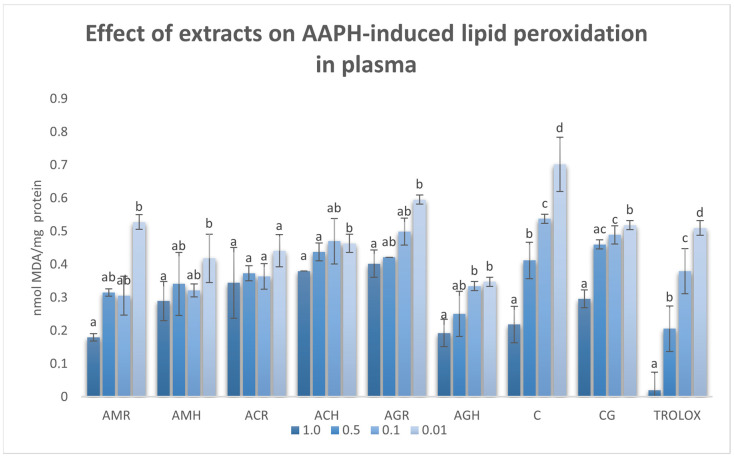
Effect of *Astragalus* extracts on AAPH-induced lipid peroxidation in plasma. Lipid peroxidation was assessed by the TBARS method and expressed as MDA content (nmol MDA/mg protein). Results are presented as mean ± SD (n = 3). AMR—*Astragalus membranaceus* root, AMH—*Astragalus membranaceus* herb, ACR—*Astragalus cicer* root, ACH—*Astragalus cicer* herb, AGR—*Astragalus glycyphyllos* root, AGH—*Astragalus glycyphyllos* herb, CG—Calycosin-7-O-*β*-D-glucoside, C—Calycosin. Different superscript lowercase letters indicate a statistically significant difference between the fractions themselves and between the fractions and control within the same column, with *p* < 0.05.

The effects of *Astragalus* extracts on AAPH-induced lipid peroxidation in plasma are presented in Figure 2. The strongest protective effect among the investigated extracts was observed for the root of AM, which reduced MDA formation to 0.18 nmol/mg protein at 1 mg/mL. Comparable activity was also noted for the herb of AG, whereas the root of AG exhibited the weakest protective effect, particularly at lower concentrations. Overall, extracts from AM and AC demonstrated moderate inhibition of plasma lipid peroxidation. Among the reference compounds, calycosin and calycosin-7-O-*β*-D-glucoside showed limited protective activity, while Trolox exhibited the strongest antioxidant effect, reducing MDA levels to 0.02 nmol/mg protein at the highest tested concentration. These results indicate that AM root possesses the greatest potential to protect plasma lipids against oxidative damage among the tested extracts.

Luo et al. reported that dietary supplementation with AM root (1% of the diet) in an in vivo model of Cashmere goats significantly improved the oxidative stability of *longissimus dorsi* muscle. Supplementation resulted in a marked reduction in lipid peroxidation, reflected by lower MDA levels compared with the control group (1.98 ± 0.83 vs. 4.81 ± 1.56 nmol/mg protein). Moreover, the treated animals exhibited significantly higher activities of the endogenous antioxidant enzymes superoxide dismutase (SOD) and catalase (CAT), indicating an enhanced antioxidant defense system and a greater resistance of muscle tissue to oxidative damage [46].

Similar observations were reported by Xu et al., who demonstrated that among different extracts of AM, the acetone extract (AE) showed the highest antioxidant potential, associated with its high phenolic content (77.79 ± 5.35 mg/g) and total antioxidant capacity (T-AOC) (0.96 ± 0.03 mM Trolox). In lipid model systems, AE most effectively reduced lipid peroxidation markers, lowering peroxide value (PO), conjugated dienes (CD) and MDA to approximately 66–69% of control values. Additionally, in fish feed, AE supplementation significantly decreased PO, CD and MDA levels, with optimal effects observed at ~6.0–6.74 g/kg, after which a plateau was reached. These results confirm that phenolic-rich *Astragalus* extracts may effectively inhibit lipid peroxidation [47].

The reduction in MDA levels observed in the present study indicates effective inhibition of lipid peroxidation and suggests a protective effect of the extracts on biological membranes. This activity is important in oxidative stress-related disorders, where lipid peroxidation drives cellular damage and disease progression.

### 3.5. Multivariate Statistical Analysis

**Figure 3:**
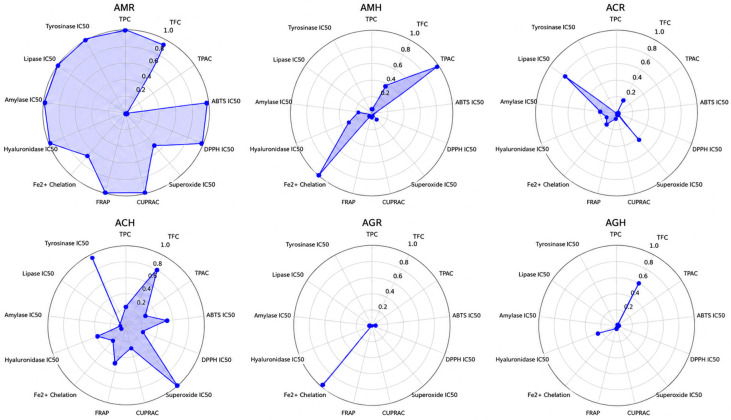
Bioactivity profiles of the investigated *Astragalus* extracts presented as radar plots based on standardized Z-scores. Higher Z-score values indicate stronger biological activity of the extracts across the analyzed parameters.

Radar plots reveal clear differences in phytochemical composition and biological activity among the analyzed extracts. AMR showed the strongest and most extensive bioactivity, characterized by the highest TPC and strong antioxidant activity in ABTS, DPPH, FRAP, and CUPRAC assays. In addition, AMR demonstrated a notable inhibitory activity against lipase and amylase, indicating the highest overall bioactive potential among the tested extracts. In contrast, AMH exhibited relatively low radical scavenging activity but higher values of TPAC and Fe^2+^ chelation, suggesting a different phenolic composition and antioxidant mechanism compared with AMR. Extracts ACR and ACH displayed intermediate activity. ACR showed moderate lipase inhibition and CUPRAC activity, whereas ACH presented slightly higher antioxidant potential, with increased TPC, CUPRAC, and superoxide scavenging. The lowest activity was observed for AGR and AGH, which showed generally weak antioxidant capacity. AGH showed moderate TPC and amylase inhibition. The results are shown in Figure 3.

**Figure 4:**
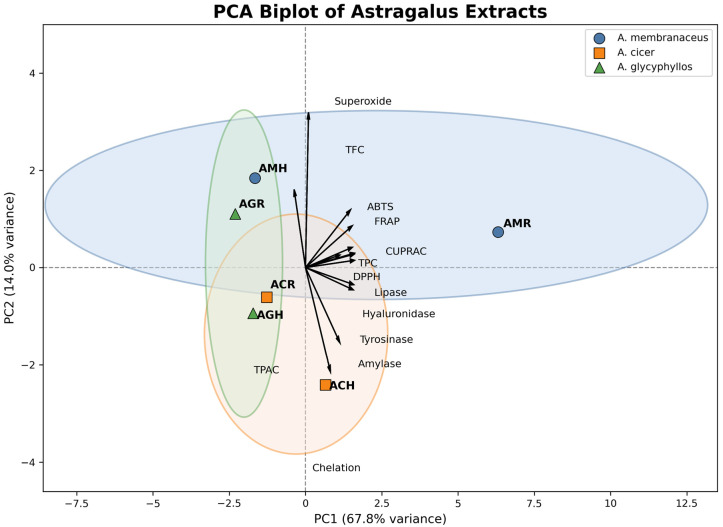
Principal component analysis (PCA) of the investigated *Astragalus* extracts based on their phenolic composition and biological activity parameters, including antioxidant and enzyme inhibitory activities.

The Principal Component Analysis (PCA) biplot shows a clear differentiation of the analyzed extracts based on their chemical composition and biological activity. The first principal component (First Principal Component—PC1, 67.8% of explained variance) separates extracts with strong antioxidant and enzymatic activity from those with lower activity, while the second component (Second Principal Component—PC2, 14.0%) mainly reflects differences related to phenolic composition and superoxide scavenging. AMR is clearly separated along the positive side of PC1, indicating a strong association with antioxidant parameters such as TPC, ABTS, DPPH, FRAP, and CUPRAC. This confirms that AMR possesses the highest antioxidant potential among the analyzed extracts and that its activity is strongly linked to phenolic compounds. AMH and AGR are positioned in the upper-left part of the plot and show a closer relationship with TFC and superoxide scavenging, suggesting that their activity may be associated with flavonoid content rather than overall phenolic concentration. ACR and AGH are located near the center of the plot, indicating moderate or balanced activity across the tested parameters. In contrast, ACH is positioned in the lower region of PC2 and is associated with enzymatic inhibition parameters such as amylase, tyrosinase, hyaluronidase, and chelation, suggesting stronger enzyme inhibitory properties compared with antioxidant activity. The results are shown in Figure 4.

**Figure 5:**
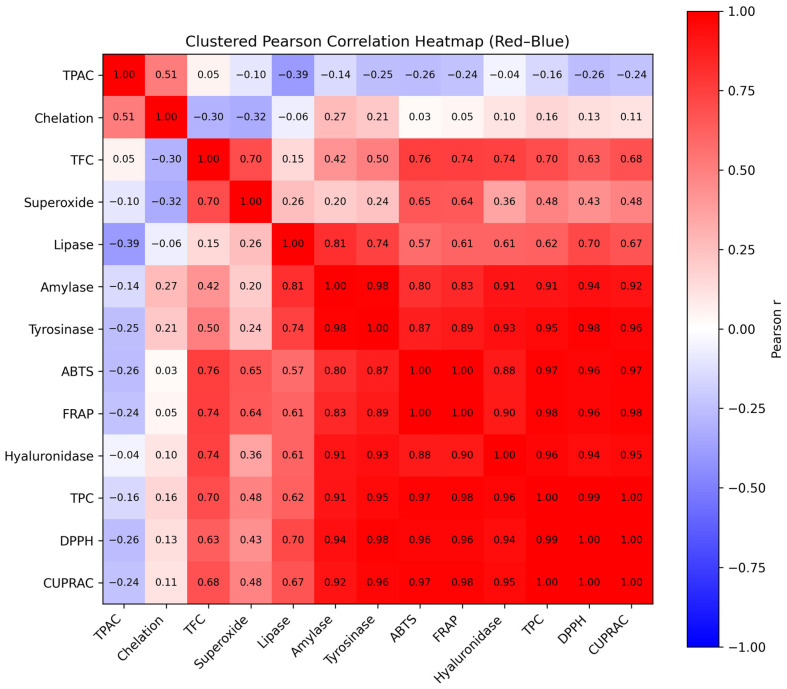
Correlation analysis (R) between antioxidant activity, enzyme inhibitory activity, and chemical composition of the analyzed *Astragalus* extracts. The color scale represents Pearson correlation coefficients, where deeper shades of blue indicate stronger positive correlations, whereas increasingly intense red colors indicate stronger negative correlations.

The clustered Pearson correlation heatmap shows strong relationships between the measured phytochemical parameters and biological activities. In general, most antioxidant and enzymatic inhibition assays are positively correlated with each other, indicating that extracts with higher bioactive compound content tend to exhibit stronger biological activity. A very strong positive correlation is observed between TPC, DPPH, CUPRAC, FRAP, and ABTS (r ≈ 0.96–1.00). This indicates that phenolic compounds play a key role in determining antioxidant capacity, as higher TPC values are associated with stronger radical scavenging and reducing activity. Similarly, strong positive correlations are also visible between DPPH, CUPRAC, FRAP, and ABTS, confirming that these assays reflect similar antioxidant mechanisms. Strong correlations are also observed among enzymatic inhibition parameters. Amylase, tyrosinase, and hyaluronidase show very high positive correlations (r ≈ 0.91–0.98), suggesting that extracts active against one enzyme are likely to exhibit inhibitory activity toward the others. Additionally, lipase inhibition is positively correlated with these enzymatic parameters and also moderately correlated with antioxidant assays. TFC shows moderate to strong positive correlations with several antioxidant parameters, particularly ABTS, FRAP, and CUPRAC, indicating that flavonoids may also contribute to the overall antioxidant activity. In contrast, TPAC and chelating activity show weaker or partially negative correlations with the majority of antioxidant parameters. This suggests that these mechanisms may be associated with different groups of compounds and may contribute to biological activity independently of the main phenolic-driven antioxidant effects. The results are shown in Figure 5.

## 4. Conclusions

The present results demonstrate clear differences in the phytochemical composition and biological activity of the analyzed *Astragalus* species occurring in Poland. Among the investigated samples, *A. membranaceus* showed significant biological activity, particularly in terms of the antioxidant and enzyme inhibitory properties, which may be associated with its high content of phenolic compounds.

Importantly, the native European species also exhibited noteworthy bioactive properties. In particular, *A. cicer* demonstrated a considerable antioxidant potential. These findings indicate that Polish representatives of the genus *Astragalus* constitute valuable sources of biologically active compounds.

Overall, the results suggest that native European species, especially *A. cicer*, may represent promising supplementary sources of natural antioxidants and could be considered as potential alternatives or complements to the widely used *A. membranaceus*. Further phytochemical and in vivo studies are warranted to confirm their pharmacological potential and to explore their possible applications in phytotherapy.

## Figures and Tables

**Table 1:** Chemical composition of extracts from *Astragalus* roots and herbs. Different superscript lowercase letters indicate statistically significant differences between samples within the same column, with *p* < 0.05.

	TPC [mg GAE/g DE]	TFC [mg QE/g DE]	TPAC [mg CAE/g DE]	Dry Extract Obtained from 10 g Raw Material [g]
AMR	199.84 ± 3.64 ^a^	43.23 ± 1.58 ^ab^	ND	2.06
AMH	135.14 ± 2.63 ^b^	32.85 ± 0.30 ^cd^	11.38 ± 0.72 ^a^	3.12
ACR	127.25 ± 1.99 ^c^	27.04 ± 3.00 ^de^	ND	1.26
ACH	150.98 ± 1.75 ^b^	44.64 ± 2.91 ^a^	2.70 ± 0.31 ^b^	2.67
AGR	128.51 ± 2.30 ^c^	22.47 ± 4.49 ^e^	ND	1.43
AGH	129.77 ± 2.23 ^b^	35.84 ± 1.90 ^bc^	ND	2.17

AMR—*Astragalus membranaceus* root, AMH—*Astragalus membranaceus* herb, ACR—*Astragalus cicer* root, ACH—*Astragalus cicer* herb, AGR—*Astragalus glycyphyllos* root, AGH—*Astragalus glycyphyllos* herb, TPC—Total Phenolic Content, TFC—Total Flavonoid Content, TPAC—Total Phenolic Acid Content.

**Table 2:** Content of polyphenolic compounds found in *Astragalus* species (mg/g of dried extract).

Content	*A. membranaceus*		*A. cicer*		*A. glycyphyllos*	
	Herb	Root	Herb	Root	Herb	Root
*p*-coumaroylquinic acids	3.67 ± 0.16	nd	nd	nd	8.87 ± 0.31	nd
Feruloylquinic acids	nd	nd	nd	nd	2.44 ± 0.03	nd
Feruloyltartaric acids	2.52 ± 0.11	nd	nd	nd	nd	nd
Caffeoylglucaric acids	nd	nd	0.98 ± 0.05	nd	nd	nd
Dicaffeoyltartaric acids	nd	108.6 ± 5.15	nd	nd	nd	nd
Caftaric acids and hexosides	nd	9.90 ± 0.43	2.57 ± 0.12	nd	nd	nd
Other phenolics	1.08 ± 0.03	2.74 ± 0.17	1.14 ± 0.01	1.04 ± 0.10	0.50 ± 0.03	det
Kaempferol derivatives	78.49 ± 3.21	nd	14.74 ± 1.02	0.16 ± 0.01	19.20 ± 1.22	
Isorhamnetin derivatives	7.26 ± 0.34	nd	1.91 ± 0.12	nd	nd	nd
Quercetin derivatives	0.14 ± 0.01	nd	3.19 ± 0.18	nd	0.42 ± 0.02	
Vitexin derivative	nd	nd	8.26 ± 0.27	nd	nd	nd
Isoorientin	nd	nd	1.64 ± 0.05	nd	nd	nd
Other flavonoids	det	nd	21.72 ± 1.62	nd	nd	nd

nd: not detected, det: detected.

**Table 3:** Anti-enzymatic activity of fractions. IC_50_ values are shown in µg/mL. Different superscript lowercase letters indicate a statistically significant difference between samples within the same column, with *p* < 0.05.

	HYAL	AMYL	LIP	Tyr
	IC_50_ [µg/mL]	IC_50_ [µg/mL]	IC_50_ [µg/mL]	IC_50_ [µg/mL]
AMR	214.51 ± 52.75 ^a^	17.78 ± 1.16 ^b^	226.7 ± 18.0 ^c^	37.32 ± 0.35 ^b^
AMH	444.79 ± 11.02 ^c^	104.01 ± 12.04 ^d^	NA	NA
ACR	634.37 ± 64.35 ^d^	102.53 ± 9.65 ^d^	288.7 ± 16.70 ^d^	NA
ACH	438.92 ± 59.47 ^c^	NA	NA	NA
AGR	849.90 ± 148.40 ^e^	>1000	NA	NA
AGH	473.30 ± 69.36 ^c^	NA	NA	NA
Astragaloside I	NA	NA	NA	NA
Astragaloside II	NA	NA	NA	NA
Astragaloside III	824.9 ± 12.40 ^e^	NA	292.80 ± 29.9 ^d^	NA
Astragaloside IV	359.90 ± 1.50 ^b^	NA	150.70 ± 11.30 ^b^	NA
Formononetin	NA	4.95 ± 0.5 ^a^	NA	NA
Formononetin-7-O-*β*-D-glucopyranoside	NA	24.93 ± 0.2 ^c^	NA	NA
Calycosin	NA	NA	NA	3.4 ± 1.2 ^a^
Calycosin-7-O-*β*-D-glucoside	NA	NA	NA	379.8 ± 11.23 ^c^
Escin	248.99 ± 24.76 ^a^			
Acarbose		0.56 ± 0.03 ^a^		
Orlistat			1.17 ± 0.04 ^a^	
Kojic acid				2.93 ± 0.17 ^a^

AMR—*Astragalus membranaceus* root, AMH—*Astragalus membranaceus* herb, ACR—*Astragalus cicer* root, ACH—*Astragalus cicer* herb, AGR—*Astragalus glycyphyllos* root, AGH—*Astragalus glycyphyllos* herb, NA—No activity.

**Table 4:** Antioxidant activity of *Astragalus* species. IC_50_ values are shown in µg/mL. The results for CUPRAC and FRAP are shown in µg/g TROLOX. The results for ferrous ion chelation are presented in %. Different superscript lowercase letters indicate a statistically significant difference between the fractions themselves and between the fractions and control within the same column, with *p* < 0.05.

	IC_50_ [µg/mL]			For 1.0 mg/mL		
	DPPH	ABTS	O_2_^●−^	Chelation of Ferrous Ion (Fe^2+^) *	CUPRAC *	FRAP *
				[%]	µg Trolox/g DE	
AMR	36.53 ± 1.22 ^a^	26.31 ± 0.03 ^b^	218.3 ± 79.3 ^c^	42.64 ± 1.14 ^d^	24.43 ± 0.62 ^c^	3.37 ± 0.23 ^c^
AMH	615.99 ± 29.98 ^e^	151.84 ± 2.79 ^g^	1118.2 ± 101.9 ^f^	58.68 ± 1.37 ^b^	1.76 ± 0.16 ^e^	0.36 ± 0.04 ^ef^
ACR	404.80 ± 120.15 ^c^	128.62 ± 3.39 ^e^	292.2 ± 7.1 ^d^	17.80 ± 2.66 ^e^	1.82 ± 0.29 ^e^	0.50 ± 0.09 ^e^
ACH	136.31± 2.03 ^b^	43.18 ± 0.74 ^c^	114.6 ± 15.0 ^b^	18.90 ± 1.74 ^e^	8.01 ± 0.55 ^d^	1.82 ± 0.18 ^d^
AGR	559.70 ± 48.84 ^d^	126.47 ± 5.20 ^d^	NA	54.29 ± 5.04 ^c^	1.31 ± 0.14 ^e^	0.37 ± 0.04 ^ef^
AGH	603.36 ± 7.35 ^e^	135.47 ± 2.77 ^f^	NA	8.57 ± 1.37 ^f^	1.67 ± 0.22 ^e^	0.29 ± 0.03 ^f^
Astragaloside I	NA	NA	NA	NA	NA	NA
Astragaloside II	NA	NA	NA	NA	NA	NA
Astragaloside III	NA	NA	NA	NA	NA	NA
Astragaloside IV	NA	NA	NA	NA	NA	NA
Formononetin	NA	NA	NA	NA	NA	NA
Formononetin-7-O-*β*-D-glucopyranoside	NA	NA	NA	NA	NA	NA
Calycosin	132.01 ± 3.0 ^b^	4.8 ± 1.2 ^a^	492.9 ± 0.9 ^e^	NA	62.16 ± 1.67 ^a^	29.33 ± 0.21 ^a^
Calycosin-7-O-*β*-D-glucoside	375.05 ± 15.0 ^c^	3.4 ± 1.2 ^a^	NA	NA	32.49 ± 1.35 ^b^	26.03 ± 0.43 ^b^
BHA	21.49 ± 0.29 ^a^	26.31 ± 0.03 ^b^				
EDTA				100 ± 0.01 ^a^		
AA			35.55 ± 0.22 ^a^			

AMR—*Astragalus membranaceus* root, AMH—*Astragalus membranaceus* herb, ACR—*Astragalus cicer* root, ACH—*Astragalus cicer* herb, AGR—*Astragalus glycyphyllos* root, AGH—*Astragalus glycyphyllos* herb, NA—No activity. * for 1 mg/mL. Different superscript lowercase letters indicate a statistically significant difference between the fractions themselves and between the fractions and control within the same column, with *p* < 0.05.

## Data Availability

The original contributions presented in this study are included in the article/Supplementary Materials. Further inquiries can be directed to the corresponding author.

## Supplementary Materials

The following supporting information can be downloaded at: https://www.mdpi.com/article/10.3390/antiox15060750/s1.

## Abbreviations

The following abbreviations are used in this manuscript:

AAPH	2,2′-azobis(2-amidinopropane) dihydrochloride
ABTS	2,2′-azinobis(3-ethylbenzothiazoline-6-sulfonic acid)
ABTS^●+^	2,2′-azinobis(3-ethylbenzothiazoline-6-sulfonic acid) radical cation
AC	*Astragalus cicer*
ACH	herb of *Astragalus cicer*
ACR	root of *Astragalus cicer*
AG	*Astragalus glycyphyllos*
AGH	herb of *Astragalus glycyphyllos*
AGR	root of *Astragalus glycyphyllos*
ANOVA	analysis of variance
AM	*Astragalus membranaceus*
AMH	herb of *Astragalus membranaceus*
AMR	root of *Astragalus membranaceus*
BHA	butylated hydroxyanisole
BSA	bovine serum albumin
BuChE	butyrylcholinesterase
CAT	catalase
CAE	caffeic acid equivalents
CD	conjugated dienes
CTAB	cetyltrimethylammonium bromide
CUPRAC	cupric reducing antioxidant capacity
DAD	diode array detector
DE	dry extract
DTNB	5,5′-dithiobis-(2-nitrobenzoic acid)
DPPH	2,2-diphenyl-1-picrylhydrazyl
DW	dry weight
EDTA	ethylenediaminetetraacetic acid
EIC	extracted ion chromatogram
ESI	electrospray ionization
FC	Folin–Ciocalteu
FRAP	ferric reducing antioxidant power
GAE	gallic acid equivalents
HA	hyaluronic acid
HCl	hydrochloric acid
HPLC	high-performance liquid chromatography
HRMS	high-resolution mass spectrometry
IC_50_	half maximal inhibitory concentration
LC–MS/MS	liquid chromatography–tandem mass spectrometry
MDA	malondialdehyde
NBT	nitro blue tetrazolium
PBS	phosphate-buffered saline
PCA	principal component analysis
PC1	first principal component
PC2	second principal component
PO	peroxide value
p-NPD	p-nitrophenyl dodecanoate
QE	quercetin equivalents
SD	standard deviation
SOD	superoxide dismutase
T-AOC	total antioxidant capacity
TBA	thiobarbituric acid
TBARS	thiobarbituric acid reactive substances
TCA	trichloroacetic acid
TFC	total flavonoid content
TOF	time-of-flight
TPAC	total phenolic acid content
TPC	total phenolic content
UHPLC	ultra-high-performance liquid chromatography
UV–Vis	ultraviolet–visible spectroscopy
