# Supplementary material for: Phenolic Profile and Antioxidant Potential of Selected European Astragalus Species: Comparative UHPLC–DAD–ESI/TOF–MS and In Vitro Study
Source: Antioxidants (Basel). 2026 Jun 13;15(6):750. doi: 10.3390/antiox15060750 (PMC13296298; doi:10.3390/antiox15060750)
Supplement: Supplementary file 1 [file antioxidants-15-00750-s001.zip › antioxidants-4344361-supplementary.pdf]

Supplementary material

Phenolic Profile and Antioxidant Potential of Selected European *Astragalus* Species: Comparative UHPLC–DAD–ESI/TOF–MS and In Vitro Study

Jakub Gębalski [1,*], Milena Gębalska [1], Ewa Kiełkowska [1], Iga Hołyńska-Iwan[2], Magdalena Wójciak[3], and Daniel Załuski[1]

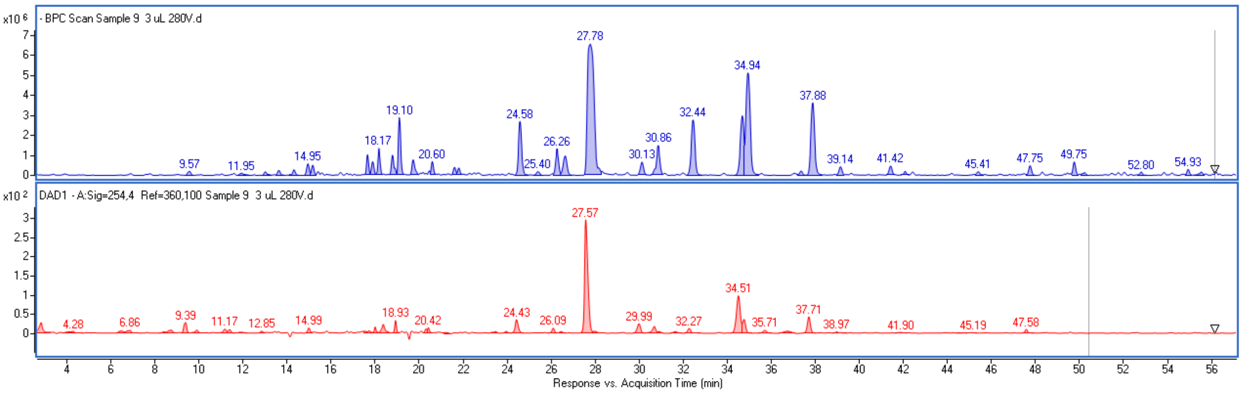

Figure S1. Representative LC–DAD-MS chromatogram of the herb of *Astragalus membranaceus*. Blue line – base peak chromatogram (BPC); red line – DAD chromatogram recorded at 254 nm.

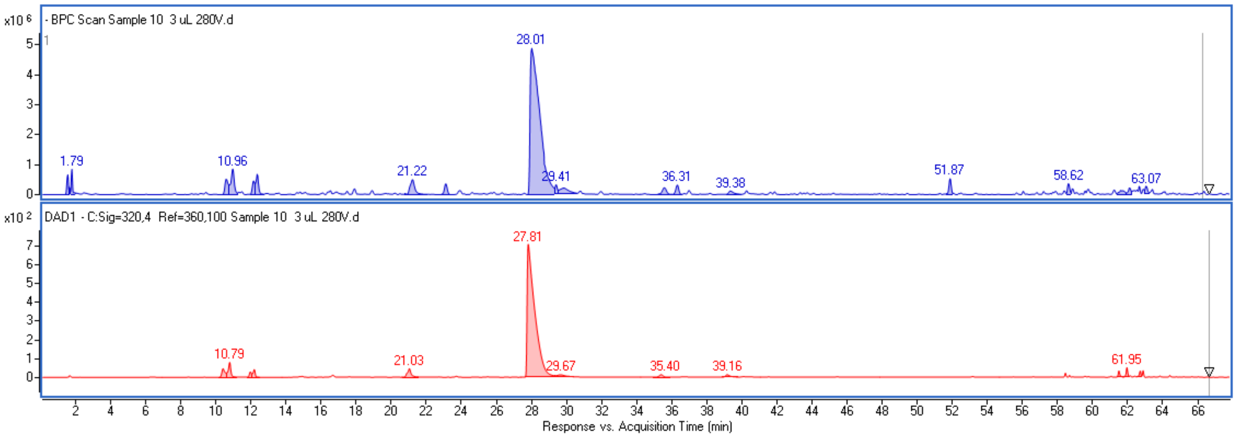

Figure S2. Representative LC–DAD-MS chromatogram of the root of *Astragalus membranaceus*. Blue line – base peak chromatogram (BPC); red line – DAD chromatogram recorded at 254 nm.

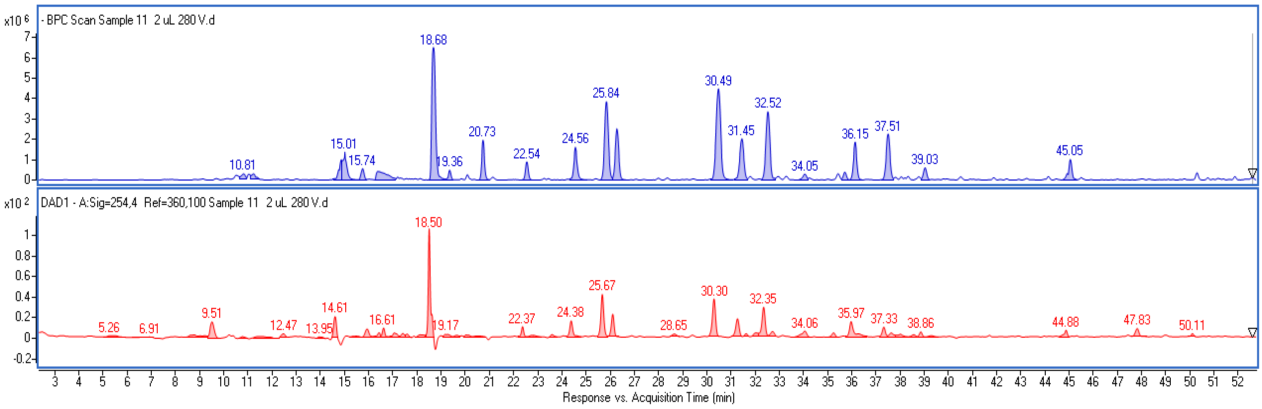

Figure S3. Representative LC–DAD-MS chromatogram of the herb of *Astragalus cicer*. Blue line – base peak chromatogram (BPC); red line – DAD chromatogram recorded at 254 nm.

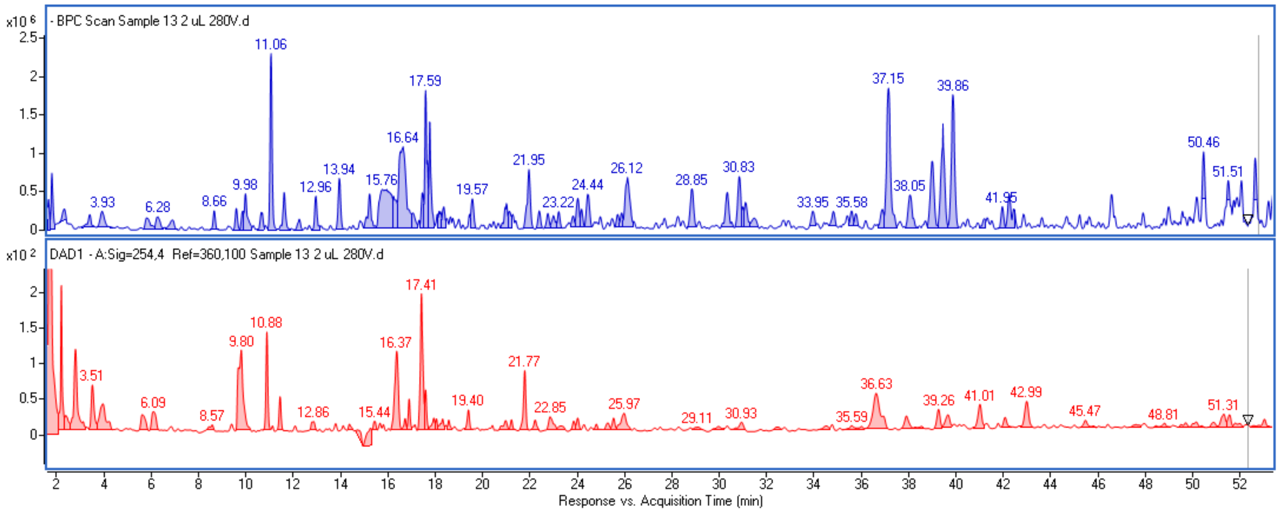

Figure S4. Representative LC–DAD-MS chromatogram of the root of *Astragalus cicer*. Blue line – base peak chromatogram (BPC); red line – DAD chromatogram recorded at 254 nm.

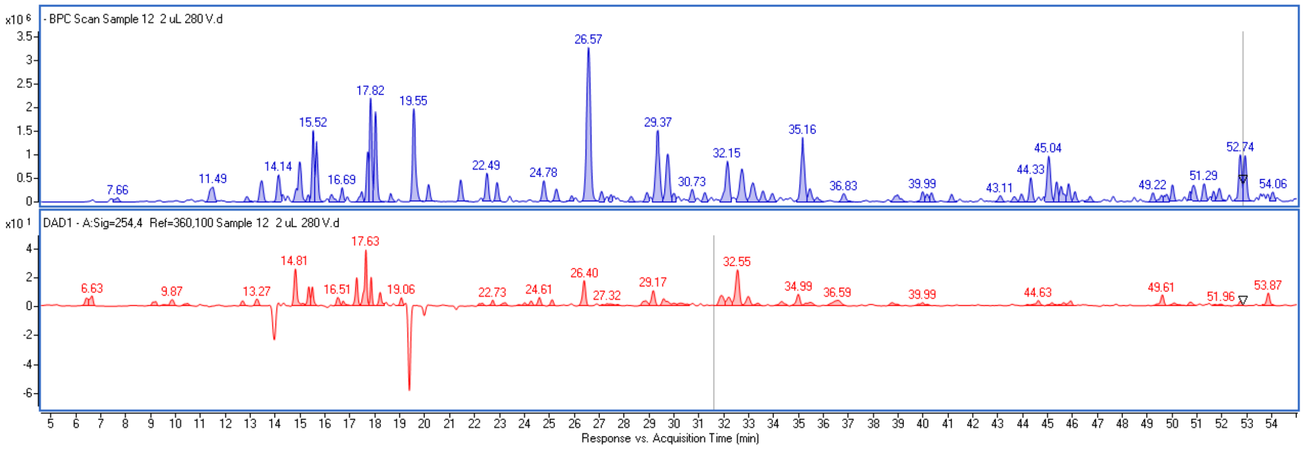

Figure S5. Representative LC–DAD-MS chromatogram of the herb of *Astragalus glycyphyllos*. Blue line – base peak chromatogram (BPC); red line – DAD chromatogram recorded at 254 nm.

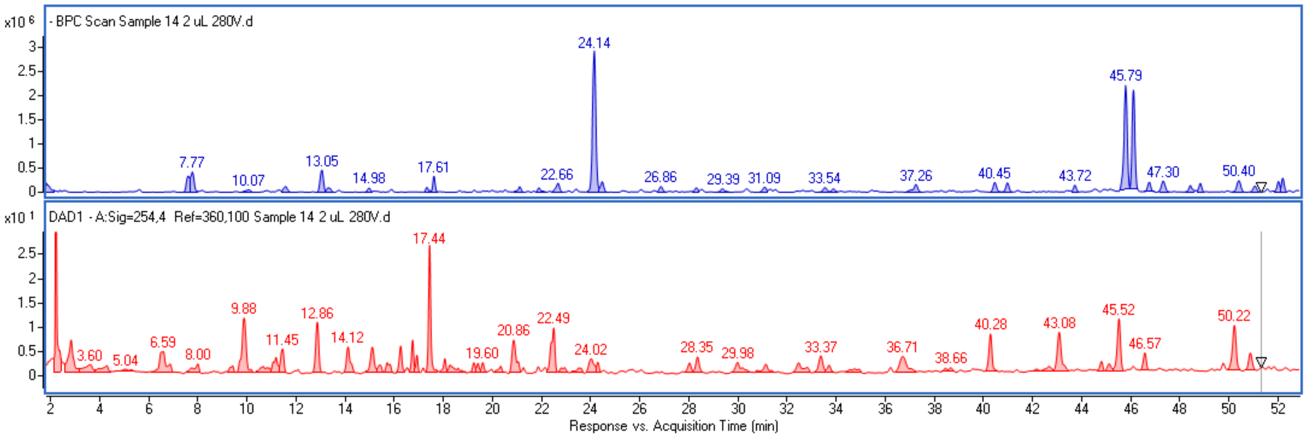

Figure S6. Representative LC–DAD-MS chromatogram of the root of *Astragalus glycyphyllos*. Blue line – base peak chromatogram (BPC); red line – DAD chromatogram recorded at 254 nm.

Sample	MS spectrum	UV-Vis spectrum
A. membranaceus herb	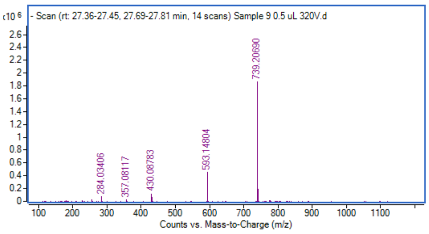	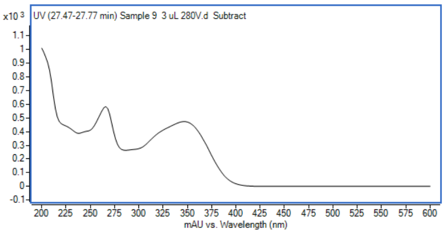
	Kaempferol derivative – estimated formula: $C_{33}H_{40}O_{19}$	
A. membranaceus herb	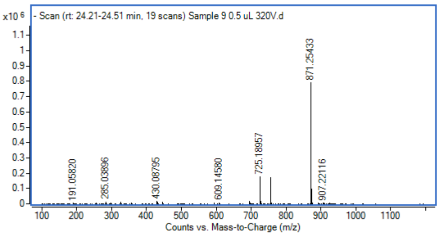	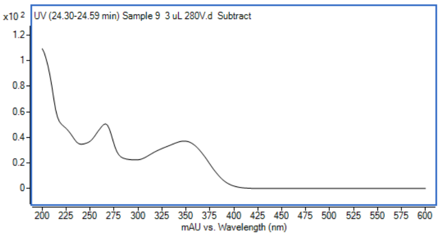
	Kaempferol derivative – estimated formula: $C_{38}H_{48}O_{23}$	
A. membranaceus root	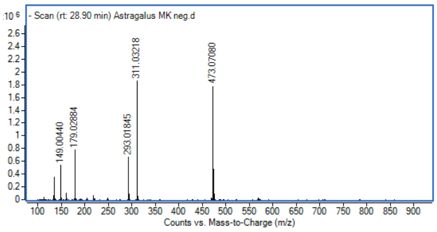	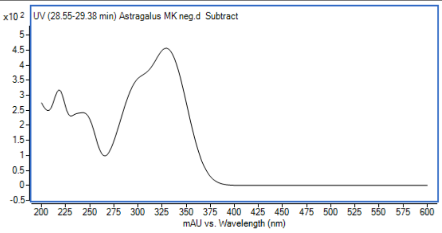
	Dicaffeoyltartaric acid (cichoric acid)	
A. cicer herb	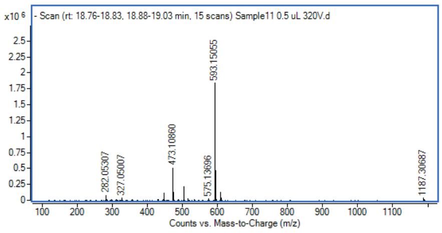	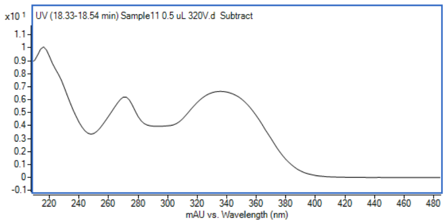
	Flavonoid - estimated formula: $C_{27}H_{30}O_{15}$	
A. cicer herb	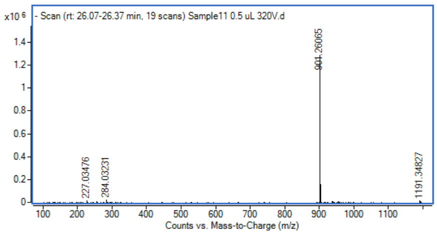	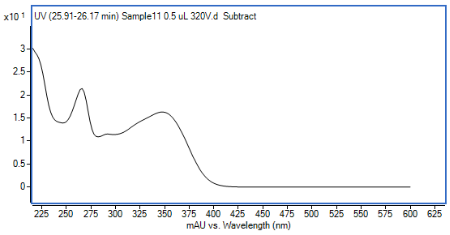
	alangiflavoside	
A. glycyphyllos herb	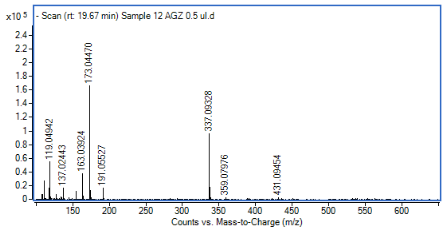	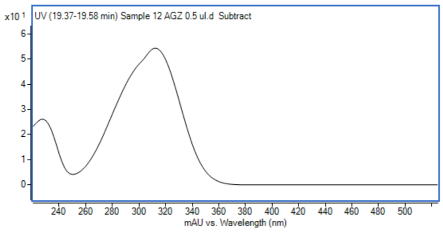
	4-*p*-coumaroylquinic acid	

Figure S7. Representative MS and UV–Vis spectra of compounds identified in the *Astragalus* extract.

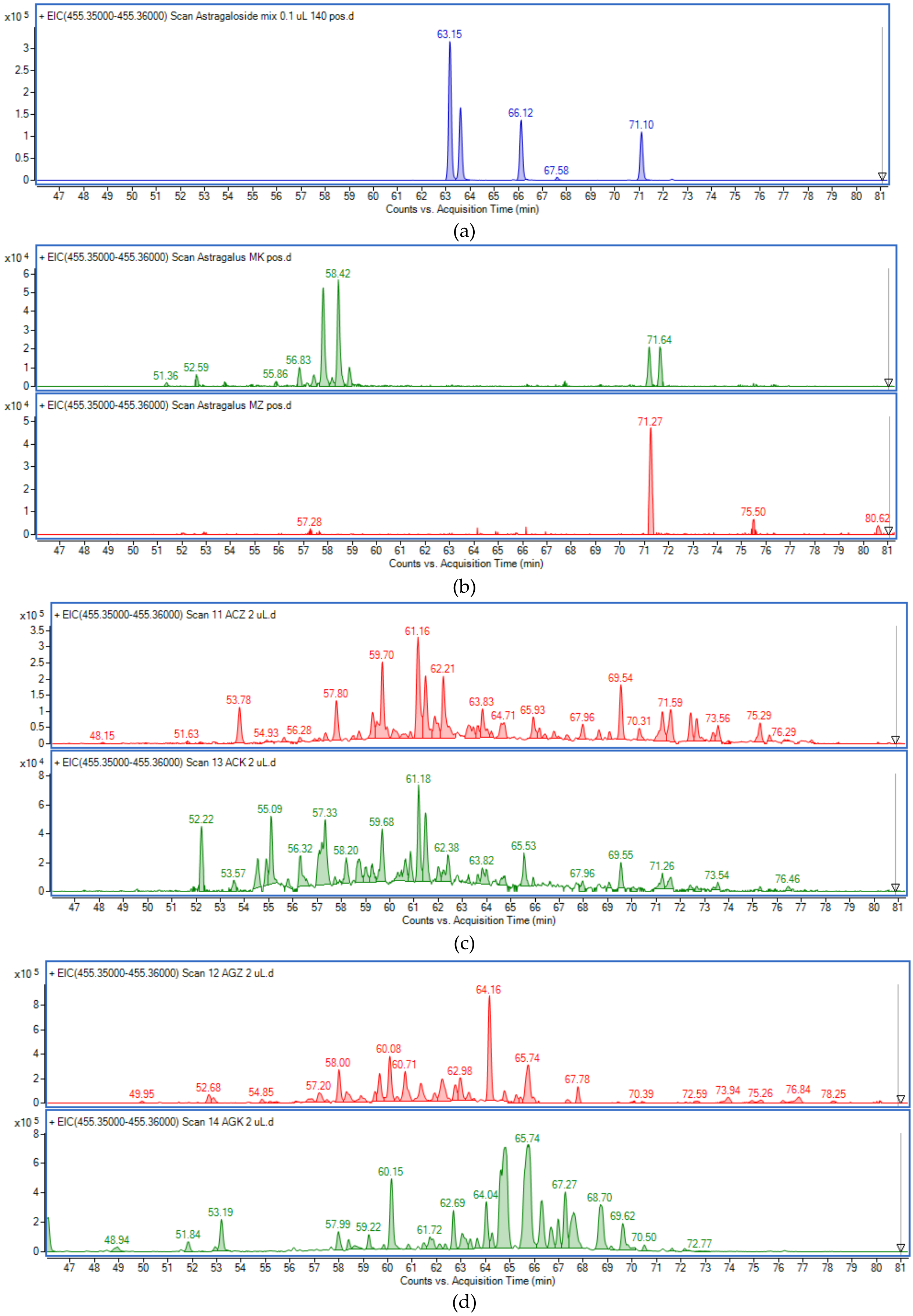

Figure S8. Extracted ion chromatogram in the mass range of m/z 455 corresponding to [aglycone + H − $H_2O]^+$, characteristic of cycloartane-type saponins. a – mixture of astragaloside I-IV, b – *A. membranaceus*, c – *A. cicer*, d - *A. glycyphyllos*.

Table S1. Mass spectrometry data of the main polyphenolic compounds found in the herb of *Astragalus membranaceus*, together with quantitative data calculated on the dried extract for compounds that were successfully quantified

Rt (min)	Observed ion mass [M-H]-/(fragments)	error (ppm)	Formula	Compound	Content (mg/g)
7.77	315.07211 (152)	-0.14	$C_{13}H_{16}O_9$	dihydroxybenzoic acid hexoside	nq
9.57	329.08707 (167)	-2.23	$C_{14}H_{18}O_9$	Vanillic acid glucose	0.77±0.02
13.02	299.07626 (137)	-3.27	$C_{13}H_{16}O_8$	Hydroxybenzoic acid glucoside	0.14±0.01
13.64	337.09209 (163,173,191)	-2.35	$C_{16}H_{18}O_8$	3-*p*-coumaroylquinic acid (I)	0.44±0.01
14.32	337.09138 (163,173,191)	-4.47	$C_{16}H_{18}O_8$	3-*p*-coumaroylquinic acid (II)*	0.89±0.03
14.95	417.10487 (152)	2.44	$C_{17}H_{22}O_{12}$	unknown	nq
15.92	367.10451 (173,193)	2.86	$C_{17}H_{20}O_9$	3-feruloylquinic acid (I)	0.14±0.01
16.43	367.10179 (173,193)	-4.53	$C_{17}H_{20}O_9$	3-feruloylquinic acid (II)	0.54±0.02
17.90	337.0918 (173,191)	-3.23	$C_{16}H_{18}O_8$	4-*p*-coumaroylquinic acid (I)	0.67±0.02
18.17	887.24667 (725)	0.44	$C_{38}H_{48}O_{24}$	Kaempferol derivative	1.34±0.06
19.10	755.20267 (593)	-1.78	$C_{33}H_{40}O_{20}$	Kaempferol derivative	2.96±0.11
19.73	337.09253 (173,191)	-1.07	$C_{16}H_{18}O_8$	4-*p*-coumaroylquinic acid (II)*	1.44±0.07
19.95	367.10169 (173,191)	-4.80	$C_{17}H_{20}O_9$	4-feruloylquinic acid (I)	0.58±0.02
20.30	337.09155 (173,191)	-3.97	$C_{16}H_{18}O_8$	5-*p*-coumaroylquinic acid (I)	0.14±0.01
20.48	367.10391 (173,191)	1.23	$C_{17}H_{20}O_9$	5-feruloylquinic acid (I)	0.13±0.01
20.60	785.21324 (623)	-1.71	$C_{34}H_{42}O_{21}$	Isorhamnetin derivatives	0.90±0.03
21.63	367.10411 (173,191)	1.78	$C_{17}H_{20}O_9$	4-feruloylquinic acid (II)	0.89±0.04
22.33	367.10332 (173,191)	-0.37	$C_{17}H_{20}O_9$	5-feruloylquinic acid (II)	0.23±0.02
22.66	337.09259 (173,191)	-0.89	$C_{16}H_{18}O_8$	5-*p*-coumaroylquinic acid (II)*	0.09±0.01
24.50	871.25426	3.32	$C_{38}H_{48}O_{23}$	Kaempferol derivative	3.94±0.12
24.58	755.20204 (725)	-2.61	$C_{33}H_{40}O_{20}$	Kaempferol derivative	0.85±0.05
26.26	901.26038 (284,593)	-1.71	$C_{39}H_{50}O_{24}$	Kaempferol derivative	1.92±0.10
26.63	475.1455 (181)	-0.45	$C_{20}H_{28}O_{13}$	unknown	nq
27.78	739.20693 (593)	-2.94	$C_{33}H_{40}O_{19}$	Kaempferol derivative	63.44±0.69
30.68	609.1449 (300)	-1.98	$C_{27}H_{30}O_{16}$	Quercetin derivative	0.14±0.01
30.86	769.21987 (623)	0.26	$C_{34}H_{42}O_{20}$	Isorhamnetin derivative	1.99±0.04
32.10	609.14779 (300)	2.76	$C_{27}H_{30}O_{16}$	Quercetin 3-*O*-rutinoside*	nq
32.44	725.19153 (284)	-2.65	$C_{32}H_{38}O_{19}$	Kaempferol derivative	0.63±0.03
34.91	593.15005 (284)	-1.93	$C_{27}H_{30}O_{15}$	Kaempferol derivative	2.98±0.11
37.09	447.09132 (284)	-4.39	$C_{21}H_{20}O_{11}$	Kaempferol hexoside	nq
37.99	623.15914 (314)	-4.20	$C_{28}H_{32}O_{16}$	Isorhamnetin derivative	4.37±0.15
38.14	593.14915 (284)	-3.44	$C_{27}H_{30}O_{15}$	Kaempferol 3-*O*-rutinoside*	0.43±0.02
39.14	623.15989 (315)	-2.99	$C_{28}H_{32}O_{16}$	Isorhamnetin derivative	nq

* - confirmed by comparison with standard; vanillic acid and hydroxybenzoic acid hexosides, *p*-coumaroylquinic acid, feruloylquinic acid as well as kaempferol, isorhamnetin, and quercetin derivatives were quantified based on calibration curves for the corresponding aglycones; nq – not quantified because lack of DAD signal or reference standard

Table S2. Mass spectrometry data of main polyphenolic compounds found in the root of *Astragalus membranaceus*. together with quantitative data calculated on the dried extract for compounds that were successfully quantified

Rt (min)	Observed ion mass [M-H]-/(fragments)	Error (ppm)	Formula	Compound	Content (mg/g)
10.96	311.04203 (179)	3.76	$C_{13}H_{12}O_9$	Caftaric acid	5.34±0.30
12.40	341.08681	-2.91	$C_{15}H_{18}O_9$	Caffeoylglucose	2.37±0.14
15.11	311.04115 (179)	0.94	$C_{13}H_{12}O_9$	Caftaric acid*	0.15±0.01
17.10	325.05808 (193)	4.83	$C_{14}H_{14}O_9$	Feruloyltartaric acid	nq
21.22	473.09317 (135,179,341)	-1.07	$C_{19}H_{22}O_{14}$	Caftaric acid glucoside	4.42±0.25
28.01	473.07082 (135,149, 179,311)	-3.65	$C_{22}H_{18}O_{12}$	Dicaffeoyltartaric acid (cichoric acid)*	107.28±5.12
29.95	473.07121 (135,149, 179,311)	-2.83	$C_{22}H_{18}O_{12}$	Dicaffeoyltartaric acid (cichoric acid)	1.31±0.04
39.38	487.08657 (135,149,179,193)	-3.34	$C_{23}H_{20}O_{12}$	Caffeoyl-feruloyltartaric acid	0.37±0.03

* - confirmed by comparison with standard; caffeoylglucose and feruloyltartaric acid were quantified based on calibration curves for caffeic acid and ferulic acid, respectively; nq – not quantified because lack of relevant standards or lack of DAD signal

Table S3. Mass spectrometry data of main polyphenolic compounds found in the herb of *Astragalus cicer* together with quantitative data calculated on the dried extract for compounds that were successfully quantified

Rt (min)	Observed ion mass [M-H]-/(fragments)	Error (ppm)	Formula	Compound	Content (mg/g)
5.35	371.06211 (209)	0.34	$C_{15}H_{16}O_{11}$	Caffeoylglucaric acid	0.18±0.01
6.00	371.06309 (209)	2.97	$C_{15}H_{16}O_{11}$	Caffeoylglucaric acid	0.11±0.01
7.10	371.06279 (209)	2.16	$C_{15}H_{16}O_{11}$	Caffeoylglucaric acid	0.09±0.01
8.33	371.06241 (209)	1.14	$C_{15}H_{16}O_{11}$	Caffeoylglucaric acid	0.21±0.01
9.47	371.06217 (209)	0.50	$C_{15}H_{16}O_{11}$	Caffeoylglucaric acid	0.19±0.01
10.45	385.07929	4.29	$C_{16}H_{18}O_{11}$	Feruloylglucaric acid	0.17±0.01
10.81	311.04121 (179)	1.14	$C_{13}H_{12}O_9$	Caftaric acid	0.72±0.03
11.45	385.07879	2.99	$C_{16}H_{18}O_{11}$	Feruloylglucaric acid	0.05±0.00
11.60	371.06248 (209)	1.33	$C_{15}H_{16}O_{11}$	Caffeoylglucaric acid	0.20±0.01
15.01	311.04095 (179)	0.30	$C_{13}H_{12}O_9$	Caftaric acid*	1.85±0.08
18.68	593.15076 (473)	-0.73	$C_{27}H_{30}O_{15}$	Flavonoid	19.96±0.89
19.06	295.0468 (163)	2.90	$C_{13}H_{12}O_8$	*p*-Coumaroyl tartaric acid	0.28±0.02
20.61	325.05691 (193)	1.24	$C_{14}H_{14}O_9$	Feruloyltartaric acid	0.32±0.02
20.73	593.14987 (473)	-2.23	$C_{27}H_{30}O_{15}$	Flavonoid	1.76±0.07
21.31	325.05682 (193)	0.96	$C_{14}H_{14}O_9$	Feruloyltartaric acid	0.34±0.02
22.54	917.25653 (300)	-0.34	$C_{39}H_{50}O_{25}$	Quercetin derivative	0.79±0.02
24.56	447.09457	2.87	$C_{21}H_{20}O_{11}$	Isoorientin*	1.64±0.05
25.84	901.26076 (284)	-1.29	$C_{39}H_{50}O_{24}$	Kaempferol derivative (alangiflavoside)	6.66±0.31
26.28	755.20444	0.56	$C_{33}H_{40}O_{20}$	Quercetin derivative	1.17±0.05
30.49	739.21034 (431)	1.67	$C_{33}H_{40}O_{19}$	Vitexin derivative	8.26±0.27
31.45	609.14672	1.00	$C_{27}H_{30}O_{16}$	Quercetin 3-*O*-rutinoside*	1.04±067

32.52	769.23285	-2.69	$C_{38}H_{42}O_{17}$	Isorhamnetin derivative	1.68±0.09
32.99	463.08999	3.86	$C_{21}H_{20}O_{12}$	Quercetin 3-*O*-glucoside*	0.19±0.01
36.15	1077.31071	1.34	$C_{49}H_{58}O_{27}$	Kaempferol derivative	3.86±0.21
37.51	593.15223	1.74	$C_{27}H_{30}O_{15}$	Kaempferol-3-*O*-rutinoside*	1.56±0.11
39.02	447.09358	0.66	$C_{21}H_{20}O_{11}$	Kaempferol-3-*O*-glucoside*	0.13±0.01
39.03	623.16265	1.43	$C_{28}H_{32}O_{16}$	Isorhamnetin 3-*O*-rutinoside*	0.23±0.02
45.05	1077.31092	1.53	$C_{49}H_{58}O_{27}$	Kaempferol derivative	2.52±0.18

* - confirmed by comparison with standard; caffeic acid, ferulic acid, kaempferol, isorhamnetin, vitexin and quercetin derivatives were quantified based on calibration curves for the corresponding aglycones. nq – not quantified because lack of DAD signal

Table S4. Mass spectrometry data of main polyphenolic compounds found in the root of *Astragalus cicer* together with quantitative data calculated on the dried extract for compounds that were successfully quantified

Rt (min)	Observed ion mass [M-H]- /(fragments)	Error (ppm)	Formula	Compound	Content (mg/g)
11.06	167.03424 (152,108)	-4.42	$C_8H_8O_4$	Isovanilic acid*	0.32±0.02
11.61	359.09918 (197,123)	2.25	$C_{15}H_{20}O_{10}$	Glucosyringic acid	0.23±0.01
15.22	295.04681 (163)	2.94	$C_{13}H_{12}O_8$	*p*-Coumaroyl tartaric acid	0.49±0.03
15.99	533.24684	3.27	$C_{21}H_{42}O_{15}$	unknown	nq
17.76	431.134431 (125)	-0.82	$C_{18}H_{24}O_{12}$	unknown	nq
17.76	447.11606 (197)	3.67	$C_{18}H_{24}O_{13}$	unknown	nq
21.95	611.16095 (167,443)	-1.31	$C_{27}H_{32}O_{16}$	unknown	nq
24.02	583.16431 (167)	-4.34	$C_{26}H_{32}O_{15}$	unknown	nq
24.44	613.17777 (197)	0.59	$C_{27}H_{34}O_{16}$	unknown	nq
31.12	447.09162 (284)	-3.72	$C_{21}H_{20}O_{11}$	Kaempferol hexoside	0.13±0.01
37.02	447.09207 (284)	-2.71	$C_{21}H_{20}O_{11}$	Kaempferol hexoside	0.03±0.00
37.15	579.20574 (417)	-4.44	$C_{28}H_{36}O_{13}$	Syringaresinol hexoside	nq
39.45	581.18639 (287,272)	-2.04	$C_{27}H_{34}O_{14}$	unknown	nq
39.86	469.1638	-3.96	$C_{29}H_{26}O_6$	unknown	nq
41.20	287.09145 (272)	-3.63	$C_{16}H_{16}O_5$	unknown	nq

* - confirmed by comparison with standard; glucosyringic acid, *p*-coumaroyl tartaric and kaempferol hexoside were quantified based on calibration curves for the corresponding aglycones; nq – not quantified because lack of relevant standards or lack of DAD signal

Table S5. Mass spectrometry data of main polyphenolic compounds found in the herb of *Astragalus glycyphyllos* together with quantitative data calculated on the dried extract for compounds that were successfully quantified

Rt (min)	Observed ion mass [M-H]- /(characteristic fragments)	Error (ppm)	Formula	Compound	Content (mg/g)
7.66	315.07341 (152)	3.97	$C_{13}H_{16}O_9$	Gentisic acid hexoside	0.09±0.01
11.07	353.08887 (179)	3.01	$C_{16}H_{18}O_9$	Neochlorogenic acid*	0.09±0.01
11.49	285.06258 (152)	3.46	$C_{12}H_{14}O_8$	Gentisic acid pentoside	0.18±0.01
13.46	337.09203 (163,173,191)	-2.55	$C_{16}H_{18}O_8$	3-*p*-coumaroylquinic acid (I)	0.92±0.03

14.14	337.09401 (163, 173,191)	3.31	$C_{16}H_{18}O_8$	3-*p*-coumaroylquinic acid (II)*	2.12±0.08
15.52	771.20163 (609,283)	3.49	$C_{33}H_{40}O_{21}$	Kaempferol derivative	1.28±0.05
15.65	917.25524 (284)	-1.47	$C_{39}H_{50}O_{25}$	Kaempferol derivative	1.66±0.06
15.74	367.10405 (173,193)	1.61	$C_{17}H_{20}O_9$	3-feruloylquinic acid (I)	0.21±0.01
16.27	367.10352 (173,193)	0.17	$C_{17}H_{20}O_9$	3-feruloylquinic acid (II)*	0.74±0.03
16.32	353.088921 (179)	3.15	$C_{16}H_{18}O_9$	chlorogenic acid	0.14±0.01
17.75	337.09212 (173,191)	-2.28	$C_{16}H_{18}O_8$	4-*p*-coumaroylquinic acid (I)	1.37±0.05
17.82	741.19636 (579,283)	2.86	$C_{32}H_{38}O_{20}$	Kaempferol derivative	3.14±0.12
17.97	887.25007 (725)	4.27	$C_{38}H_{48}O_{24}$	Kaempferol derivative	1.84±0.06
19.57	337.09329 (173,191)	1.18	$C_{16}H_{18}O_8$	4-*p*-coumaroylquinic acid (II)*	3.67±0.13
19.70	367.10231 (173,191)	-3.11	$C_{17}H_{20}O_9$	4-feruloylquinic acid (I)	0.26±0.02
20.14	337.09299 (173,191)	0.29	$C_{16}H_{18}O_8$	5-*p*-coumaroylquinic acid (I)	0.49±0.02
21.44	367.10398 (173,191)	1.42	$C_{17}H_{20}O_9$	4-feruloylquinic acid (II)	1.03±0.09
22.25	367.10405 (173,191)	1.61	$C_{17}H_{20}O_9$	5-feruloylquinic acid (I)	0.19±0.01
22.49	337.092113 (173,191)	-2.30	$C_{16}H_{18}O_8$	5-*p*-coumaroylquinic acid (II)	0.29±0.01
24.68	367.10397 (173,191)	1.40	$C_{17}H_{20}O_9$	5-feruloylquinic acid (II)	nq
26.57	755.20539 (284)	1.82	$C_{33}H_{40}O_{20}$	Kaempferol derivative	2.66±0.11
29.37	609.14643 (284)	0.53	$C_{27}H_{30}O_{16}$	Kaempferol dihexoside	1.39±0.05
29.75	739.20792 (284)	-1.60	$C_{33}H_{40}O_{19}$	Kaempferol derivative	0.72±0.06
32.15	725.19103 (284)	-3.34	$C_{32}H_{38}O_{19}$	Kaempferol derivative	1.25±0.07
33.09	463.08665	-3.34	$C_{21}H_{20}O_{12}$	Quercetin 3-*O*-glucoside*	0.42±0.02
33.95	917.2328 (755)	-3.17	$C_{42}H_{46}O_{23}$	Kaempferol derivative	nq
34.30	917.23295 (755)	-3.01	$C_{42}H_{46}O_{23}$	Kaempferol derivative	nq
35.00	917.23307 (755)	-2.88	$C_{42}H_{46}O_{23}$	Kaempferol derivative	nq
35.11	579.13359 (284)	-3.37	$C_{26}H_{28}O_{15}$	Kaempferol derivative	0.79±0.04
36.74	447.09256 (284)	-1.62	$C_{21}H_{20}O_{11}$	Kaempferol hexoside	0.23±0.02
38.96	447.09433 (284)	2.33	$C_{21}H_{20}O_{11}$	Kaempferol 3-*O*-glucoside*	0.25±0.01
39.99	901.23661 (284)	-4.64	$C_{42}H_{46}O_{22}$	Kaempferol derivative	0.47±0.03
44.33	901.23806 (284)	-3.03	$C_{42}H_{46}O_{22}$	Kaempferol derivative	0.42±0.03
45.36	931.24795 (284)	-3.66	$C_{43}H_{48}O_{23}$	Kaempferol derivative	0.55±0.04
45.84	901.23856 (284)	-3.48	$C_{42}H_{46}O_{22}$	Kaempferol derivative	1.03±0.08
45.93	931.24818 (284)	-3.41	$C_{43}H_{48}O_{23}$	Kaempferol derivative	0.54±0.04
49.44	755.17985 (284)	-4.02	$C_{36}H_{36}O_{18}$	Kaempferol derivative	0.31±0.02
50.91	755.17997 (284)	-3.86	$C_{36}H_{36}O_{18}$	Kaempferol derivative	0.67±0.05
52.74	669.42333 (715)	2.08	$C_{36}H_{62}O_{11}$	unknown	nq
52.92	801.46455 (847)	0.44	$C_{41}H_{70}O_{15}$	unknown	nq

* - confirmed by comparison with standard; *p*-coumaroylquinic acid, feruloylquinic acid, and kaempferol derivatives were quantified based on calibration curves for the corresponding aglycones. nq – not quantified because lack of DAD signal

Table S6. Mass spectrometry data of main polyphenolic compounds found in the root of *Astragalus glycyphyllos* together with quantitative data calculated on the dried extract for compounds that were successfully quantified

Rt (min)	Observed ion mass [M-H]-/(fragments)	Error (ppm)	Formula	Compound	Content (μg/g)
7.77	315.07117 (152)	-3.12	$C_{13}H_{16}O_9$	Gentisic acid glucoside	87.6±5.3
11.42	285.06244 (152)	2.97	$C_{12}H_{14}O_8$	Gentisic acid pentoside	17.1±1.5
11.59	359.09787 (197,123)	-1.39	$C_{15}H_{20}O_{10}$	Glucosyringic acid	164.1±11.2
13.05	299.07685 (137)	-1.30	$C_{13}H_{16}O_8$	Hydroxybenzoic acid hexoside	198.9±15.6
17.61	431.11803 (125)	-3.40	$C_{18}H_{24}O_{12}$	unknown	nq
24.14	471.16443	-3.44	$C_{25}H_{28}O_9$	unknown	nq
26.86	609.14318 (271)	-4.80	$C_{27}H_{30}O_{16}$	unknown	nq
33.59	435.0931	-0.42	$C_{20}H_{20}O_{11}$	unknown	nq
37.05	579.21101 (417)	4.65	$C_{28}H_{36}O_{13}$	Syringaresinol hexoside	nq
45.79	771.45515 (817)	1.97	$C_{40}H_{68}O_{14}$	unknown	nq
50.40	461.10733 (299)	-3.47	$C_{22}H_{22}O_{11}$	Flavonoid	nq
51.05	299.05529	-2.74	$C_{16}H_{12}O_6$	Flavonoid	nq
53.23	771.45275 (817)	-1.14	$C_{40}H_{68}O_{14}$	unknown	nq

* - confirmed by comparison with standard; nq – not quantified because lack of relevant standards or lack of DAD signal

Table S7. Validation data used for quantitative analysis

**Analyte**	**Wavelength**	**Concentration range**	**Equation**	**Correlation**
Vanillic acid	260 nm	1.0-10 μg/mL	26.926x - 1.4025	0.9994
Gentisic acid	325 nm	0.1-2.0 μg/mL	19.636x - 2.91	0.9988
Chlorogenic acid	325 nm	0.1-2.0 μg/mL	17.53x - 1.3194	0.9985
p-hydroxybenzoic acid	260 nm	0.1-2.0 μg/mL	13.2775x - 1.4316	0.9986
p-coumaric acid	310 nm	0.2-10 μg/mL	54.535x - 0.1263	0.9996
Ferulic acid	325 nm	0.2-10 μg/mL	60.435x - 0.4222	0.9996
Caffeic acid	325 nm	1.0-10 μg/mL	44.611x + 7.5276	0.9999
Caftaric acid	325 nm	1.0-10 μg/mL	32.392x - 4.775	0.9992
Dicaffeoyltartaric acid	325 nm	25-250 μg/mL	16.733x + 161.67	0.9994
		1.0-10 μg/mL	19.81x + 29.679	0.9986
Kaempferol 3-*O*-rutinoside	350 nm	1.0-10 μg/mL	10.772x - 2.2439	0.9991
		20-200 μg/mL	9.7303x + 26.232	0.9997
Kaempferol 3-O-glucoside	350 nm	0.1-2.0 μg/mL	16.487x - 1.1282	0.9987
Quercetin 3-*O*-rutinoside	350 nm	0.1-10 μg/mL	11.638x - 1.0462	0.9989
Quercetin 3-O-glucoside	350 nm	0.1-2.0 μg/mL	20.355x - 0.8902	0.9986
Isorhamnetin 3-*O*-rutinoside	350 nm	1.0-10 μg/mL	13.816x - 4.3123	0.9996
Isoorientin	350 nm	1.0-10 μg/mL	16.69x - 3.125	0.9992
Vitexin	350 nm	2.0-20 μg/mL	18.715x - 2.375	0.9996

Table S8. Isoflavones found in *Astragalus* extract (μg/g of dried extract)

TR (min.)	Compound	*Astragalus membranaceus*		*Astragalus cicer*		*Astragalus glycyphyllos*	
		herb	root	herb	root	herb	root
28.17	Calycosin-7-*O*-β-D-glucoside	nd	30.87±2.11	3.38±031	16.36±1.12	3.83±0.34	13.18±1.02
43.35	Formononetin 7-*O*-β-D-glucoside	nd	100.28±4.56	27.19±1.67	3.10±0.21	4.70±0.31	68.34±3.25
52.04	Calycosin	7.32±0.65	24.88±1.57	0.74±0.07	2.22±0.18	2.42±0.19	12.50±1.01
62.75	Formononetin	121.12±6.02	183.78±9.21	6.99±0.70	2.01±0.18	7.61±0.65	233.52±11.51

Compounds were determined using extracted ion chromatograms (EIC) in the mass range characteristic of the compounds.
